# Modelling Dolphin Distribution to Inform Future Spatial Conservation Decisions in a Marine Protected Area

**DOI:** 10.1038/s41598-018-34095-2

**Published:** 2018-10-23

**Authors:** Cecilia Passadore, Luciana M. Möller, Fernando Diaz-Aguirre, Guido J. Parra

**Affiliations:** 1 Cetacean Ecology, Behaviour and Evolution Lab, College of Science and Engineering, Flinders University, South Australia, 5001 Australia; 2 Molecular Ecology Lab, College of Science and Engineering, Flinders University, South Australia, 5001 Australia

## Abstract

As marine predators experience increasing anthropogenic pressures, there is an urgent need to understand their distribution and their drivers to inform spatial conservation planning. We used an ensemble modelling approach to investigate the spatio-temporal distribution of southern Australian bottlenose dolphins (*Tursiops* cf. *australis*) in relation to a variety of ecogeographical and anthropogenic variables in Coffin Bay, Thorny Passage Marine Park, South Australia. Further, we evaluated the overlap between current spatial management measures and important dolphin habitat. Dolphins showed no distinct seasonal shifts in distribution patterns. Models of the entire study area indicate that zones of high probability of dolphin occurrence were located mainly within the inner area of Coffin Bay. In the inner area, zones with high probability of dolphin occurrence were associated with shallow waters (2–4 m and 7–10 m) and located within 1,000 m from land and 2,500 m from oyster farms. The multi-modal response curve of depth in the models likely shows how the different dolphin communities in Coffin Bay occupy different embayments characterized by distinct depth patterns. The majority of areas of high (>0.6) probability of dolphin occurrence are outside sanctuary zones where multiple human activities are allowed. The inner area of Coffin Bay is an important area of year-round habitat suitability for dolphins. Our results can inform future spatial conservation decisions and improve protection of important dolphin habitat.

## Introduction

Information on how different environmental and anthropogenic variables affect the distribution of species is fundamental for understanding their ecology and guiding spatial conservation planning^1,2^. The presence and distribution of marine top predators, such as dolphins, has been linked to a variety of abiotic and biotic factors, which are usually linked to the distribution of their prey, predators and conspecifics^3^. Human activities such as boating, fishing activities and aquaculture can affect dolphin behaviour and ultimately also influence their distribution patterns^4–6^. Species distribution models (SDM) provide a useful analytical framework to investigate the environmental and anthropogenic factors affecting species distribution^1,2,7^. Such information can help elucidate which areas constitute important habitat for a species and where potential conflicts with human activities may occur^8^.

In the marine environment, coastal ecosystems are the most heavily impacted by human activities^9^. Marine top predators such as whales and dolphins are particularly susceptible to human stressors because of their life-history traits (i.e. late maturity, low reproductive rate and long life span^10^) and some of the most at risk species occur in coastal areas. Several coastal dolphin populations, especially those with high levels of site fidelity and restricted ranging patterns, are at risk due to pressures such as habitat degradation and loss, by-catch, prey depletion, tourism, pollution, among others^11–16^. The decline of dolphins’ numbers due to anthropogenic disturbances can be reverted if areas of high abundance and suitable habitats are identified, and appropriate spatial conservation planning and management measures (including enforcement) are established to diminish anthropogenic impacts within those areas^17–19^.

Australia has the world’s largest representative network of marine parks covering 3.3 million km^2^ (36%) of its marine environment. Despite this protection, the waters surrounding Australia’s coastline are increasingly threatened by human activities and several areas across northern, western and southern Australia have been identified as global hotspots of marine mammal extinction risk^20^. Furthermore, few studies have focused on investigating whether Australia’s marine protected areas are adequately protecting marine mammals^21^. In South Australia (SA), increasing coastal zone development, coastal pollution, aquaculture and fishery interactions, threaten the viability of dolphin populations^22–25^. Our understanding of the magnitude of these problems and ability to provide effective management solutions to them is hindered by the lack of spatially explicit data on dolphin distribution and anthropogenic threats. There is an urgent need for this information as zoning of all SA’s marine parks is schedule for review in 2022, and there is strong commitment from wildlife agencies to ensure that the marine planning process includes the conservation needs of marine top predators such as dolphins.

The bottlenose dolphin (*Tursiops* sp.) is a cosmopolitan marine top predator, extensively distributed in temperate and tropical waters around the world. Currently there are two widely accepted species within the genus, the common bottlenose dolphin (*T. truncatus*) and the Indo-Pacific bottlenose dolphin (*T. aduncus*). *T. truncatus* is considered by the IUCN Red List of Threatened Species as Least Concern^26^, while *T. aduncus* is classified as Data Deficient^27^. Recently, a potential new species was described for coastal waters of southern Australia, the Burrunan dolphin (*Tursiops australis*)^28^. The taxonomy of this putative new species is still contentious^29,30^, therefore we refer to them here as southern Australian bottlenose dolphins (*Tursiops* cf. *australis*) or SABD. SABD appear to form small, resident and genetically differentiated populations^31^, and population structuring may be occurring at small spatial scales in relation to environmental factors (e.g. location of oceanographic front^32^). So far, six populations of SABD have been identified spread over ~2500 km of coastline based on molecular markers^28,31,33,34^. These populations are exposed to different environmental conditions and anthropogenic activities, but little is known about how these may influence their distribution patterns. Studies in Gulf Saint Vincent, SA, showed that the distribution patterns of SABD are influenced by a variety of ecogeographic variables, likely linked to prey distribution and availability, such as bare sand habitat in the Port River estuary and Barker Inlet^35^, and water depth, benthic habitat type and slope along Adelaide’s metropolitan coast^36^. Both studies identified priority areas for dolphin conservation along SA’s coast and highlighted the need for future studies to evaluate the influence of human activities (e.g. vessel traffic, fishing, and ports) on dolphin distribution.

**Figure 1:**
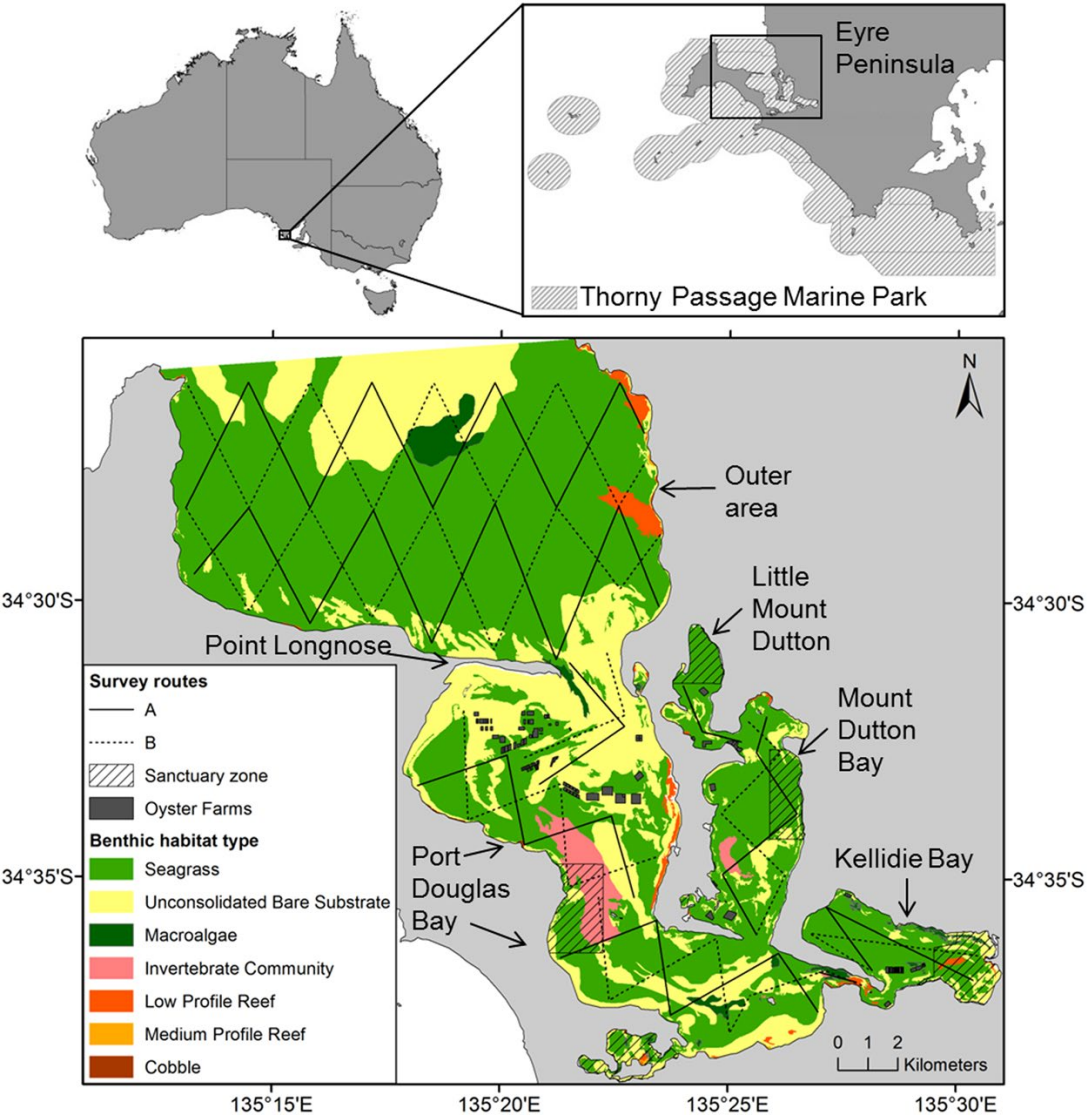
Location of Coffin Bay within the Thorny Passage Marine Park, Eyre Peninsula, South Australia. Study area showing the zig-zag transect layout (Survey routes A and B) used to cover the outer and the inner areas of Coffin Bay, oyster farms and sanctuary zones. Colours as indicated in the legend represent the different types of benthic habitats (Database provided by the Department of Environment, Water and Natural Resources, South Australian Government).

The largest population of SABD (n = 306, 95% CI: 291–323) studied to date inhabits Coffin Bay, a small embayment (263 km^2^) located within the Thorny Passage Marine Park, Eyre Peninsula, SA^37^. Coffin Bay is an heterogeneous ecosystem with two distinctive areas, the outer area, which is exposed to the oceanographic conditions of the Southern Ocean, and the inner area, which is a shallow inverse estuary consisting of a variety of habitats across several interconnected embayments^38,39^. The inner area sustains a high density of dolphins (1.57–1.7 individuals/km^2^), most of them residents, likely favoured by the high biological productivity and the apparent low predation risk in this area^37,40^. The status of these dolphins, i.e. whether the population size is stable or not, is unknown^37^. About 6% of Coffin Bay waters are currently classified as sanctuary zones (i.e. areas of high conservation value where only low-impact recreation activities are allowed, but motorized water sports and fishing are prohibited), while the rest of the bay is zoned as a multiple use marine park where several human activities are allowed (e.g., boating, oyster aquaculture, recreational fishing^41,42^). The local human population around Coffin Bay is relatively small (*c.a*. 500 people) but increases to *c.a*. 4,000 people during the peak tourist season (end-December to February, and Easter)^43^. The main human activities occurring in Coffin Bay waters that could have detrimental effects to the local dolphin population are aquaculture and vessel traffic^43^. The inner area of Coffin Bay is home to SA’s leading Pacific oyster aquaculture industry with several areas designated for farming (Fig. 1). Furthermore, the bay attracts substantial power boating activity, particularly during the summer and Easter tourism seasons, including recreational fishing, fishing charters, and cruises for sightseeing and to see and taste oysters in the farms, and to a smaller degree, for dolphin watching^43^. Elsewhere, shellfish aquaculture has been associated with dolphins’ habitat loss because of a decrease in occurrence and distribution of dolphins in areas around farms^44–47^. Vessel traffic is also known to affect dolphins’ behaviour in the short-term^48–50^, can cause injuries or death due to collisions^51^, and could lead to population declines or abandonment of habitat in the long term^6,52^. Despite the importance of Coffin Bay for SABD, the current lack of information about their distribution patterns in relation to environmental conditions and human activities hampers the identification of important habitats and the potential impacts of these threats. Understanding how aquaculture and vessel traffic may affect dolphins’ distribution patterns is crucial for improving future decision-making regarding the zoning of multiple-use MPAs in SA.

In this study, we used an ensemble of SDMs^53^ to assess the spatio-temporal distribution of SABD in relation to a variety of ecogeographical and anthropogenic variables in Coffin Bay, SA. The aim was to identify areas of high probability of dolphin occurrence, gain insights into the habitat requirements of the species and evaluate the relevance of the current sanctuary zones to the protection of dolphins within this MPA. The results improve our understanding of the spatial ecology of the species, illustrate the importance of considering both environmental as well as anthropogenic factors in SDMs, and support future spatial conservation planning in southern Australia.

## Results

Between September 2013 and October 2015, we encountered 620 groups of dolphins (587 and 33 in the inner and outer areas, respectively) over 144 days of surveys. Survey effort and number of dolphin groups sighted varied between seasons, and between the inner and the outer areas of Coffin Bay (Supplementary Appendix 1, Table S1 and Fig. S1). Overall, the highest survey effort and number of dolphin sightings occurred within the inner area (Supplementary Appendix 1, Table S1 and Fig. S1).

### Dolphin occurrence across Coffin Bay

**Figure 2:**
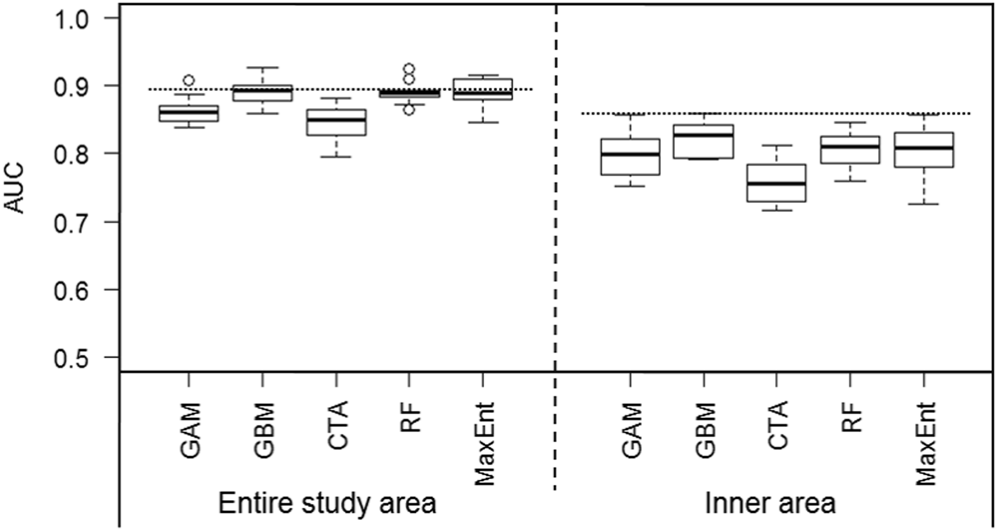
Performance of species distribution models built with datasets of the entire study area (left) and the inner area (right) of Coffin Bay. Box-plots for the model accuracy (AUC: area under the curve of the receiver operating characteristics plot) of the 10 cross-validation runs of each modelling algorithm (GAM: generalised additive model; GBM: generalised boosted model; CTA: classification tree analysis; RF: random forest; and MaxEnt: maximum entropy), and dotted line indicating the predictive performance (AUC) of ensemble models for each dataset. Values of AUC ≥ 0.7 indicate that the model predictive performance is moderate to excellent.

**Figure 3:**
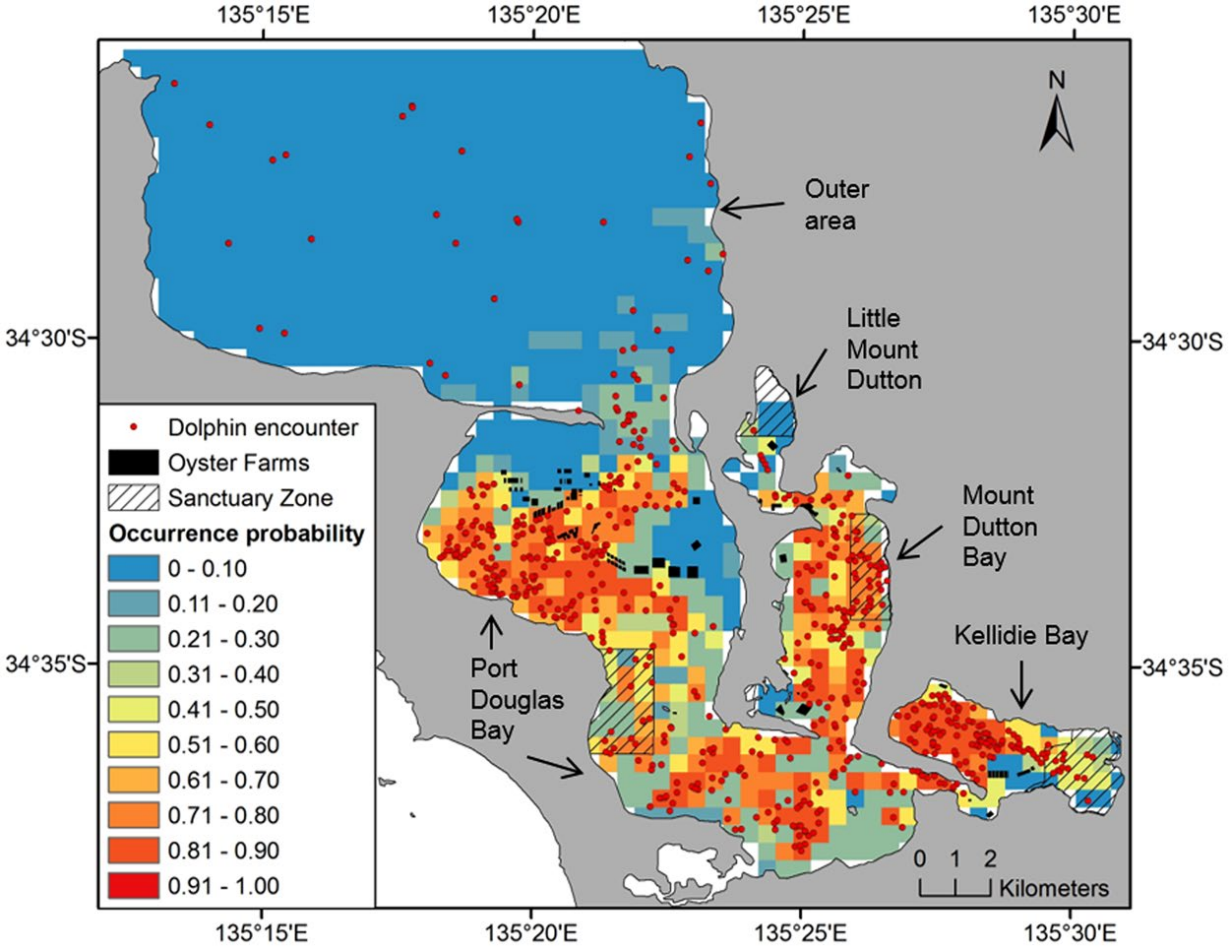
Ensemble model of SABD (*Tursiops* cf. a*ustralis*) probability of occurrence in Coffin Bay for the overall study period (September 2013 – October 2015). The coloured shading, as detailed in the legend, represents probability of dolphin occurrence.

When considering data across the entire study area and study period, collinearity was detected between distance to farm and distance to sanctuary zone (r = 0.92), and depth and distance to land (r = 0.72). After running ‘vifstep’, distance to farm and to land were discarded from modelling. Thus, the remaining explanatory variables included in SDMs for the whole study area were habitat type, distance to sanctuary zones, and water depth (Table 1). Single SDMs performance varied from moderate to excellent (AUC median = 0.88; range: 0.79–0.93), and ensemble models (AUC = 0.90) had better performance than most single SDMs (Fig. 2). The most important variable in all single SDMs was distance to sanctuary zone, followed by water depth (Table 1). The probability of dolphin occurrence was higher in areas between 500 and 5,000 m from sanctuary zones, and where water depth was shallower than 15 m, with peaks in dolphin occurrence at water depths of 2–4 m and 7–10 m (Supplementary Appendix 2, Fig. S4). These ranges of distance to sanctuary zones and water depth are characteristic of the inner area only (Supplementary Appendix 1, Fig. S2). Accordingly, the ensemble model of the whole study area predicted high dolphin presence mainly within the inner area of Coffin Bay (Fig. 3). Similarly, seasonal models indicated that the most important predictor of dolphin presence was distance to sanctuary zone (or distance to farm), and predicted areas of high probability of dolphin in the inner area (see Appendix 4).

**Table 1:** Importance of ecogeographical and anthropogenic variables used in SDMs of SABD (*Tursiops* cf. a*ustralis*) for the whole study area and for the inner area of Coffin Bay: GAM = generalised additive model; GBM = generalised boosted model; CTA = classification tree analysis; RF = random forest; and MaxEnt = maximum entropy. Variable importance is presented as the mean value over the 10 runs of each single modelling algorithm, and as the mean of means amongst them. Explanatory variables of greatest influence (values closest to one) are highlighted in bold. (NOTE: Values are presented only for those non-correlated variables included in each model).

Study area	Models	Habitat type	Distance to sanctuary zone	Water depth	Distance to land^a^	Distance to oyster farm^a^
Whole	GAM	0.036	**0.803**	0.394	—	—
	GBM	0.003	**0.855**	0.426	—	—
	CTA	0.002	**0.881**	0.417	—	—
	RF	0.033	**0.710**	0.447	—	—
	MaxEnt	0.009	**0.875**	0.247	—	—
	Mean of means	0.016	**0.825**	0.386	—	—
Inner	GAM	0.057	0.091	**0.806**	0.113	0.173
	GBM	0.006	0.057	**0.817**	0.076	0.106
	CTA	0.006	0.041	**0.935**	0.044	0.065
	RF	0.018	0.119	**0.663**	0.182	0.150
	MaxEnt	0.019	0.065	**0.732**	0.131	0.134
	Mean of means	0.021	0.075	**0.791**	0.109	0.126

^a^Distance to land and to oyster farm were excluded from the modelling procedure for the whole study area as they showed collinearity with depth and distance to land, respectively.

### Dolphin occurrence in inner area

**Figure 4:**
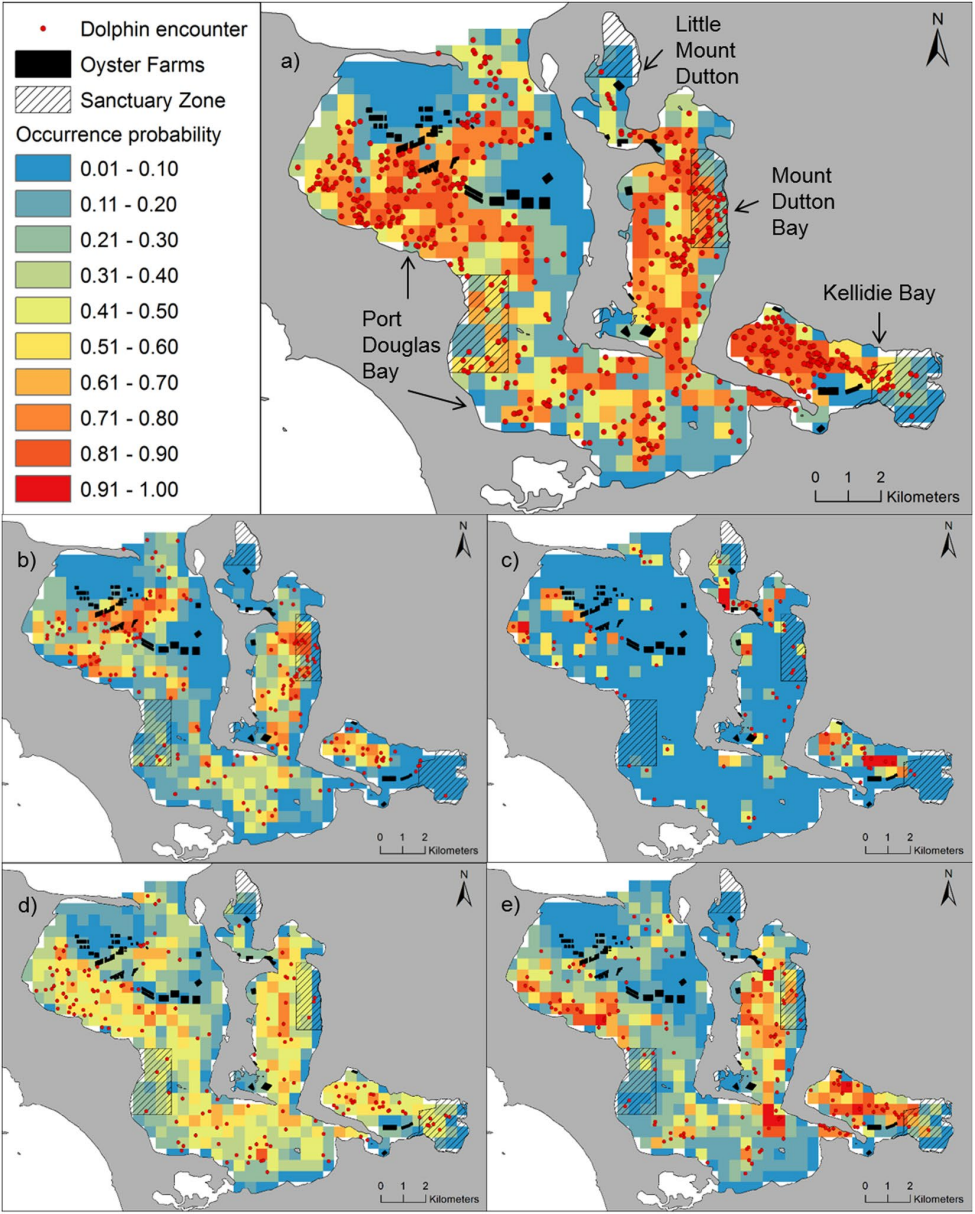
Ensemble models of SABD (*Tursiops* cf. a*ustralis*) probability of occurrence in the inner area of Coffin Bay for: (**a**) over the entire study period; (**b**) spring; (**c**) summer; (**d**) autumn; and (**d**) winter. Colours as shown in the legend indicate the probability of occurrence of dolphins.

We found no collinearity between the explanatory variables considered for SDMs of the inner area (r < 0.26 and VIF < 1.3 for all combinations of variables), thus all variables were retained for analysis. Single SDMs performance varied from moderate to excellent (AUC median = 0.80; range: 0.72–0.86), and ensemble models outperformed all single SDMs (AUC = 0.86; Fig. 2). The most important variable affecting the distribution of dolphins in the inner area over the entire study period was water depth, followed by distance to oyster farms and to land (Table 1). The probability of dolphin occurrence was higher in areas deeper than 2 m, within a distance of 2,500 m from oyster farms, and within 1,000 m from land (Supplementary Appendix 2, Fig. S5). The ensemble model predicted high dolphin presence mainly in the north-west part of Port Douglas Bay, in some parts of Mount Dutton Bay, and the western part of Kellidie Bay (Fig. 4a).

### Seasonal dolphin occurrence in inner area

Collinearity was found between water visibility and depth in every season (r > 0.74). After running ‘vifcor’, water visibility was discarded from seasonal models. In autumn, pH and salinity also showed high collinearity (r = −0.74), and thus salinity was discarded from the models after running ‘vifstep’ (Table 2). Single seasonal SDMs of the inner area showed poor (AUC < 0.7) to moderate performance (0.7 ≤ AUC < 0.9) (Supplementary Appendix 3, Fig. S6), thus some models were excluded from ensembles. The ensemble models outperformed all single SDMs in every season (Supplementary Appendix 3, Fig. S6). Most seasonal SDMs identified water depth as the most important variable, followed by distance to land (Table 2); which is concordant with results of overall models for the inner area (Table 1). Exceptions included two algorithms for spring and three for autumn that had distance to land as the most important variable, and two algorithms for summer that identified pH as an important variable (Table 2). Response curves of SDMs showed variability among SDMs (see examples in Supplementary Appendix 3, Fig. S7). Among seasonal ensemble predictions, summer exhibited the lowest probability of dolphin presence (Fig. 4b–e). In summer, the highest probabilities of dolphin occurred in the central part of Kellidie Bay, and the northern part of Mount Dutton Bay and the entrance to Little Mount Dutton (Fig. 4c). In the remaining seasons, the highest probability of dolphins were in areas where water depth exceeds 2 m including the western sector of Kellidie Bay, the central part of Mount Dutton Bay and around the farms of Port Douglas Bay (Fig. 4b,d and e).

**Table 2:** Importance of ecogeographical and anthropogenic variables for SABD (*Tursiops* cf. a*ustralis*) in the inner area of Coffin Bay by season, using five types of models: generalised additive model (GAM), generalised boosted model (GBM), classification tree analysis (CTA), random forest (RF) and maximum entropy (MaxEnt). Variable importance is presented as the mean value over the 10 runs of each single modelling algorithm, and as the mean of means amongst them. Explanatory variables of greatest influence (values closest to one) are highlighted in bold. (NOTE: Values are presented only for those non-correlated variables included in each model).

Season	Model	Habitat type	Distance to sanctuary zone	Water depth	Distance to land	Distance to oyster farm	Vessel encounter rate	Salinity^a^	Sea surface temperature	pH
Spring	GAM	0.088	0.129	0.350	**0.390**	0.018	0.061	0.086	0.093	0.193
	GBM	0.008	0.090	**0.421**	0.318	0.051	0.035	0.020	0.047	0.111
	CTA	0.017	0.172	**0.477**	0.460	0.120	0.134	0.072	0.125	0.206
	RF	0.009	0.088	**0.320**	0.243	0.053	0.040	0.023	0.061	0.084
	MaxEnt	0.013	0.033	0.339	**0.476**	0.024	0.021	0.032	0.037	0.020
	Mean of means	0.027	0.102	**0.381**	0.377	0.053	0.058	0.047	0.073	0.123
Summer	GAM	0.184	0.102	**0.427**	0.275	0.189	0.038	0.060	0.171	0.280
	GBM	0.043	0.058	0.181	0.160	0.201	0.029	0.004	0.105	**0.350**
	CTA	0.146	0.026	0.185	0.218	0.500	0.055	0.000	0.083	**0.551**
	RF	0.048	0.065	**0.157**	0.111	0.149	0.021	0.012	0.101	0.163
	MaxEnt	0.234	0.053	**0.315**	0.195	0.101	0.065	0.013	0.041	0.077
	Mean of means	0.131	0.061	**0.253**	0.192	0.228	0.042	0.018	0.100	0.284
Autumn	GAM	0.258	0.031	0.320	**0.438**	0.053	0.132	—	0.018	0.054
	GBM	0.052	0.105	**0.291**	0.258	0.088	0.108	—	0.030	0.063
	CTA	0.078	0.337	**0.527**	0.306	0.269	0.210	—	0.073	0.115
	RF	0.045	0.091	0.171	**0.208**	0.081	0.080	—	0.050	0.062
	MaxEnt	0.091	0.021	0.247	**0.350**	0.070	0.132	—	0.031	0.043
	Mean of means	0.105	0.117	**0.311**	**0.312**	0.112	0.132	—	0.040	0.067
Winter	GAM	0.151	0.162	**0.406**	0.307	0.091	0.089	0.167	0.032	0.101
	GBM	0.007	0.059	**0.427**	0.160	0.052	0.027	0.227	0.011	0.120
	CTA	0.008	0.044	**0.515**	0.250	0.117	0.026	0.372	0.007	0.264
	RF	0.019	0.061	**0.284**	0.153	0.057	0.028	0.133	0.026	0.097
	MaxEnt	0.053	0.089	**0.389**	0.276	0.104	0.055	0.053	0.013	0.028
	Mean of means	0.048	0.083	**0.404**	0.229	0.084	0.045	0.191	0.018	0.122

^a^Salinity was excluded from the modelling procedure for autumn as it showed collinearity with pH.

### Dolphin occurrence and sanctuary zones

According to ensemble models’ predictions, the probability of dolphin occurrence in sanctuary zones over the whole study period ranged from 0.06 to 0.83 (Fig. 4). Amongst all sanctuaries, the one located in Mount Dutton Bay had the highest probability (mean ± SD = 0.52 ± 0.28) of dolphin occurrence (Fig. 4; Table 3). The seasonal mean probabilities of dolphin occurrence were below 0.5 for all sanctuaries (Table 3).

**Table 3:** Probability of occurrence of SABD (*Tursiops* cf. a*ustralis*) predicted by the inner area’s ensemble models in sanctuary zones (SZ) of Coffin Bay.

Sanctuary zone	Area (km^b^)	No. grids	Overall (Mean ± SD)	Spring (Mean ± SD)	Summer (Mean ± SD)	Autumn (Mean ± SD)	Winter (Mean ± SD)
Kellidie	4.5	18	0.25 ± 0.23	0.05 ± 0.02	0.07 ± 0.19	0.26 ± 0.18	0.32 ± 0.3
Little Mount Dutton	3.1	5	0.11 ± 0.03	0.04 ± 0.01	0.18 ± 0.19	0.17 ± 0.11	0.07 ± 0.02
Mount Dutton	3.1	20	0.52 ± 0.28	0.38 ± 0.32	0.08 ± 0.14	0.31 ± 0.18	0.33 ± 0.26
Port Douglas	4.8	21	0.43 ± 0.23	0.2 ± 0.1	0.04 ± 0.03	0.32 ± 0.11	0.15 ± 0.08
Outside	107.5	431	0.41 ± 0.28	0.25 ± 0.23	0.12 ± 0.21	0.34 ± 0.19	0.29 ± 0.25

Overall and seasonal probability values (mean ± SD) of all the grids falling in each SZ (i.e. in Kellidie, Mount Dutton, Little Mount Dutton and Port Douglas) or outside them are shown.

## Discussion

Effective management of wildlife populations requires sound knowledge of species distributions and associated threats. Here, we used an ensemble modelling approach to determine the spatio-temporal distribution patterns of SABD in Coffin Bay, a heterogeneous ecosystem located within a multiple use marine park in SA. Ensemble modelling provided a robust approach for evaluating the importance of ecogeographical and anthropogenic factors influencing dolphin distribution patterns, and identifying important areas of dolphin occurrence. Distance to sanctuary zones and water depth were the most important variables influencing dolphins’ probability of occurrence over Coffin Bay. High probability of dolphin occurrence was predicted almost exclusively for the inner area of Coffin Bay, which is consistent with the high density of dolphins recorded for this area^37^, and indicates that the inner area represents an important habitat for SABD. Models of the inner area showed that dolphins favoured waters greater than 2 m deep, within a distance of 1,000 m from land and 2,500 m from oyster farms. Despite the seasonality in environmental conditions and anthropogenic activities, the most important explanatory variables influencing dolphin distribution were similar across seasons and there were no significant shifts in dolphin distribution patterns. Overall, we found that areas with the highest probability of dolphin presence were located in three different embayments within the inner area: Mount Dutton, Kellidie and Port Douglas. Most areas of highest dolphin probability (>0.6) were located outside sanctuary zones.

Dolphin distribution is influenced by prey distribution and predation risk^54–56^. Therefore, characteristics of the habitat such as water depth, distance to coast, salinity, sea surface temperature, among others, are usually used as proxies of prey availability in SDMs because they are related to oceanographic processes that enhance local productivity e.g.,^36,57,58^. SABD favoured the waters of the inner area of Coffin Bay. Shallow, sheltered, inverse estuaries, such as the inner area of Coffin Bay, are usually highly productive systems^59^ that can sustain high densities of fish and top predators like dolphins. The total nutrient loads in the inner area of Coffin Bay are higher than those of outer area^60^, and it is likely that this enhances the productivity in the former resulting in higher abundance of prey. Several fish and cephalopods known to be part of the diet of bottlenose dolphins in SA^61^, use Coffin Bay as a nursery and feeding area^42^. Furthermore, it is likely that differences in predation risk between the inner and outer area of Coffin Bay may also influence dolphin occurrence patterns in the study area. White sharks (*Carchharodon carcharias*), one of the predators of dolphins along SA’s coast^62^, can be found close to shore in <5 m depth, but they seem to prefer continental shelf waters <100 m depth^63^. The shallow waters of the inner area and the narrow connection with the outer area may restrict the use of the former by sharks, thus resulting in lower predation risk in the inner area. To explicitly test these hypotheses, future studies need to incorporate additional variables into SDMs such as chlorophyll *a* or net primary production, as well as the presence and abundance of prey and predators.

In temperate regions, dolphins can display seasonality in their distribution patterns as they follow changes in prey abundance and availability, which are driven by seasonal changes in water conditions^36,64^. Although Coffin Bay is exposed to pronounced spatial and temporal variability in environmental conditions (Supplementary Appendix 2, Fig. S3), dolphin distribution patterns showed no major changes with season. This temporal stability in the distribution patterns of SABD indicates year-round habitat suitability in the inner area of Coffin Bay, suggesting that the availability of prey in the inner area is enough to fulfil dolphins needs year round, contrary to what is observed along the Adelaide coast^36^. The Adelaide metropolitan coast is an open environment, likely less productive than Coffin Bay, where the abundance of SABD varies throughout the year, and individuals show varying levels of site fidelity and residency^65^.

Apart from ecological factors, the social structure of animal populations can also influence individual patterns of space use^66–68^. Two social communities of SABD (each one with at least 70 individuals) occur in the inner area of Coffin Bay, one in the Port Douglas area and another one in Mount Dutton and Kellidie Bays^69^. Furthermore, the space use patterns of resident dolphins of the inner area are characterized by strong site fidelity, small representative ranges (<33.5 km^2^) and restricted movements to a single embayment^40^. The multi-modal response curves observed for the applied models likely reflect the dolphin community preferences for different embayments within Coffin Bay and their respective characteristics^69^. The plateau of occurrence probability observed at 2–4 m depth may relate to the dolphin community inhabiting Kellidie and Mount Dutton Bays, where the mean depth of this bays are 2 and 4 m, respectively; and the plateau at 7–10 m may relate to the community occurring in Port Douglas Bay, where depth can reach up to 11 m (Supplementary Appendix 1, Fig. S2b). Thus, the areas of high probability of dolphin occurrence identified here likely reflect the interaction among ecological and social factors.

Anthropogenic activities such as aquaculture and vessel traffic are known to affect dolphin distribution patterns (e.g.,^4–6^). Dolphins’ response to aquaculture activities is variable and complex. Some studies elsewhere showed that dolphins were attracted to areas with aquaculture^44,70,71^, while others showed that dolphins were less likely to go into areas where aquaculture was occurring, even though farms were located in habitats with characteristics favored by dolphins^47^. In Coffin Bay, oyster farms are located in shallow areas less than 2 m deep, while dolphins seem to prefer waters greater than 2 m deep. Whether dolphins have been displaced from areas now occupied by oyster farms, since the farms were established, is unknown. In general, shellfish aquaculture is known to increase nitrogen levels into the ecosystem altering local ecology, especially in areas where tidal and other flushing is minimal^72^. The inner area of Coffin Bay has slow flushing^39^ and high nutrient loads^60^. A trophic mass-balance model used to estimate the potential effects of finfish aquaculture in Aranci Bay, Sardinia, Italy, showed increased nutrient loading into aquaculture farm areas, followed by increases in biomass of fish and top predators, such as bottlenose dolphins^71^. Thus, dolphins favouring areas within 500 to 2,500 m from oyster farms in Coffin Bay is likely in response to higher nutrients and a potential increase in prey abundance in the proximity of farms. Further studies on dolphin diet and prey distribution within the study area are needed to test this hypothesis.

Although the influence of encounter rate of vessels was not as strong as other variables in explaining the distribution of dolphins, response curves showed that the probability of dolphin presence decreased as vessel encounter rates increased (Supplementary Appendix 3, Fig. S7), suggesting that dolphins in Coffin Bay tend to occur in areas with lower vessel traffic. Future behavioural research and long-term monitoring of this population would help elucidate whether dolphins’ behaviour is affected by the presence of oyster farms or vessels, and if management measures are required to prevent potential long-term consequences.

Our findings highlight areas with high probability of dolphins (>0.6) located in three different embayments within the inner area of Coffin Bay (i.e. Kellidie, Mount Dutton and Port Douglas, see Fig. 4a). Sanctuary zones cover areas with low (<0.3) to moderate (0.31–0.6) probability of dolphin’s presence in Kellidie and Port Douglas Bays, and relatively high probability in Mount Dutton Bay. However, in general, areas with the highest probability of dolphin presence are outside the sanctuary zones, in multiple use areas where dolphins are exposed to a variety of anthropogenic threats including vessel traffic, recreational fishing and oyster farming. Dolphins favoured areas close to oyster farms and such proximity can put them under risk of entanglement with aquaculture gear, which may cause injuries or death^47,51,70^. The farming system used in Coffin Bay uses structures that result in debris washing up on beaches^38^, including poles, baskets, rubber bands and plastic clips. During this study, four calves were observed swimming with rubber bands entangled around their necks, while two of them were still alive at the end of the study, the remaining two were presumed dead (unpublished data). The expansion of current or the establishment of new oyster farms in Coffin Bay should take into account the areas of high dolphin presence identified here to minimize interactions with aquaculture equipment and potential displacement of dolphins from important habitats.

Marine mammals are considered as ‘species of ecological value’ in the management plan of the Thorny Passage Marine Park^73^. However, there are no specific management arrangements to protect SABD. The high density of dolphins inhabiting Coffin Bay^37^, and the findings presented here should encourage the integration of the species into the monitoring program and zoning arrangements of this park. We recommend the areas of high dolphin presence identified here as priority areas for dolphin conservation and for the implementation of vessel traffic, aquaculture and fishing regulations.

## Methods

### Study area

Coffin Bay is part of the Thorny Passage Marine Park, SA (Fig. 1). Coffin Bay’s benthic habitats are mainly seagrass beds, followed by unconsolidated bare substrate, invertebrate community, low profile reef, macroalgae, cobble and medium profile reef (Fig. 1). The bay is divided by a spit of land into an inner (~123 km^2^) and an outer area (~155 km^2^), and water exchange between these two areas is restricted through a narrow (2 km) opening^39^. The inner area is a shallow (mean depth ~2.5 m with tides of approx. 1.3 m) system that consists of several interconnected bays (e.g. Port Douglas, Mount Dutton and Kellidie^38,39^). This area is considered an inverse estuary because evaporation rates exceeds precipitation during the austral summer resulting in hypersaline waters; while in winter salinity is diluted because of freshwater inputs^39,41^. The outer area connects the waters from the inner area to the Great Australian Bight, and is influenced by oceanographic features of the Southern Ocean^38^. In the outer area productivity is low during winter; however, a nearby summer-autumn (February and March) upwelling brings cold, nutrient-rich water to the surface^74,75^. In the study area, especially in the internal bays, marked seasonal fluctuations are observed in water conditions such as sea surface temperature (SST) and salinity^39^.

### Survey design and data collection

Boat-based line-transect surveys were conducted between September 2013 and October 2015 to collect location data on dolphins and vessels. Surveys were conducted along two alternative equal-spaced zigzag transect routes^76^ covering a range of environmental conditions (*e.g*., depth, distance to shore, temperature, salinity) and human activities (*e.g*., location of aquaculture farms, distribution of vessels). To complete a single route in the inner and outer area, it took 2–4 and 2–3 days of surveys, respectively. Transects covered 85.5 km^2^ in the inner area and 154.1 km^2^ in the outer area of Coffin Bay. Surveys were done during daylight hours, at an average speed of 15 km/hr and under good weather conditions (*i.e*. Beaufort state ≤3, good-average visibility, no rain or fog, swell height <1 m). Once a route was completely surveyed in each area, we started with the alternate route on the next day of survey. During surveys, an observer on each side of the boat searched continuously for dolphins and vessels from −5° to 90° degrees of the transect with Fujinon 7 × 50 binoculars or the naked eye. All observers were trained in dolphin observation techniques to reduce observer bias in dolphin detection and group size estimation. A group of dolphins was defined as all animals seen within a radius of approx. 100 m^77^. Whenever a group of dolphins was sighted the position of the research vessel on the transect was recorded with a GPS, and search effort was suspended to approach the group within 10–20 m, and record their location using a GPS and group size. Whenever an operating power vessel (i.e. with people on board who were either navigating or fishing), or group of vessels (defined as ≥2 vessels encountered within a radius of 100 m), was sighted on a transect the following data were gathered: GPS position on transect, number of vessels, horizontal sighting angle, and downward angle (in reticles) to vessel (or to the centre of the group), measured with the binoculars compass and reticles, respectively. This information was used to derive the position of vessels using formulae proposed by Lerczak and Hobbs^78^. Data on environmental variables (water depth, sea surface temperature, turbidity, salinity and pH) were collected *in situ* at the location of every group of dolphins encountered, every 2 km along the transect line, and at the beginning and end of each transect leg. An YSI Professional Plus handheld multiparameter was used to record sea surface temperature (accuracy ± 0.2 °C), salinity (accuracy ± 0.1 ppt) and pH (accuracy ± 0.2 units); turbidity was measured using a Secchi disc; and depth was recorded using the boat’s depth sounder.

### Data analysis

A Geographic Information System (GIS) in ArcMap 10.3.1 (ESRI) was used to create spatial layers of all response (dolphin presence-absence) and ecogeographic and anthropogenic explanatory variables (Table 4) at 500 × 500 m grid cell resolution. The location of dolphin groups and survey tracks were imported into ArcMap to create a binary presence-absence grid of dolphins while taking into account survey effort. A grid layer of survey effort (km^2^) was generated by adding a 500 m buffer (average distance to which dolphins could be reliably observed from the boat) on either side of the transect surveyed. Survey coverage was quantified for the entire study period and per season by calculating the total amount of area surveyed on-effort within each grid during each time period.

**Table 4:** List of anthropogenic and ecogeographic variables considered for modelling the presence-absence of SABD (*Tursiops* cf. *australis*) in Coffin Bay. For each variable we show its classification, the type (i.e. categorical or numeric) and range of values, and the data source. It is also indicated if a particular variable was used in overall and/or seasonal models.

Classification	Explanatory variables	Type: Values	Data source	Included in models	
				Overall	Seasonal
Anthropogenic	Distance to sanctuary zone	Numeric, continuous: 0–21,188 m	NatureMaps^a^	*Yes*	*Yes*
	Distance to oyster farm	Numeric, continuous: 0–15,558 m	PIRSA^b^	*Yes*	*Yes*
	Distance to land	Numeric, continuous: 0–6,756 m	NatureMaps^a^	*Yes*	*Yes*
	Vessels encounter rate^c^	Numeric, continuous: 0–700	*In situ*	No	Yes
Ecogeographic	Benthic habitat type	Categorical, categories: seagrass beds, unconsolidated bare substrate, low profile coral reefs, macroalgae, invertebrate community, cobble and medium profile coral reefs	NatureMaps^a^	Yes	Yes
	Water depth	Numeric, continuous: 0–36 m	*In situ*	Yes	Yes
	Salinity (surface)^c^	Numeric, continuous: 30–47 PSU	*In situ*	No	Yes
	Sea surface temperature^c^	Numeric, continuous: 11.5–25.9 °C	*In situ*	No	Yes
	Water visibility^c^	Numeric, continuous: 0–16.5 m	*In situ*	No	Yes
	pH^c^	Numeric, continuous: 7.7–9.0	*In situ*	No	Yes

^a^Layers on coastline, habitat type, and zoning of marine parks were obtained from the NatureMaps provided by the South Australian Government (Department of Environment, Water and Natural Resources, available at https://data.environment.sa.gov.au/NatureMaps/Pages/default.aspx).

^b^The location of aquaculture leasing zones (hereafter referred as oyster farms), were obtained from the Spatial Information Services of Primary Industries and Resources SA (PIRSA).

^c^These variables vary temporally (see Results) and were pooled by austral season and used only in the seasonal SDMs.

Obtaining data on true absences for mobile species is difficult^79^. In dolphin studies, false absences can occur due to observer error (visibility bias), when animals are underwater and remain undetected (availability bias), or if survey effort is not high enough to reliably cover the study area^79–81^. Including false absences in models that require presence-absence data can produce inaccurate predictions of species distribution^82^. As true absence data were not available, for presence-absence models we generated inferred absence data (pseudo-absences) by incorporating survey effort in the definition of absences^82,83^. Similarly to previous studies, we defined pseudo-absence cells based on areas with highest survey effort^36,84^. For each area (inner and outer), we calculated the mean survey effort per grid. After this, grids in the inner and outer areas with survey effort higher than the mean per area, and with no presence of dolphins, were considered pseudo-absences. This definition of pseudo-absence allows us to assume that selected grids are as close to ‘true’ absences as possible, since they were surveyed several times during the study period without dolphin detections. We generated the same number of pseudo-absences as available presences, which results in an equal weighting of presences and pseudo-absences in the species–habitat models, a procedure that has been shown to perform well for a variety of SDM algorithms^81^. For Maxent, which is a presence-only approach that requires background samples of the environment^85^, we used as background data those grid cells with environmental data that were surveyed in a given data set (i.e. entire Coffin Bay or inner area) and period (i.e. entire study period or seasons), regardless of amount of effort.

Ecogeographical and anthropogenic explanatory variables were selected based on the availability of data and published evidence suggesting that they could potentially affect the presence of bottlenose dolphins or their prey (e.g.,^5,36,57,58,86,87^). Each 500 × 500 m grid within the study area was characterised by each ecogeographical and anthropogenic explanatory variable considered in this study (Table 1). The distance to sanctuary zones, oyster farms, and to land was measured using the Euclidean distance function in ArcMap. The benthic habitat type of each grid cell was assigned as the category (Table 1) covering the greatest proportion of each cell. To generate raster layers of the environmental data collected *in situ* (*i.e*. water depth, SST, salinity, water visibility and pH), the point data were interpolated in ArcMap using the Ordinary Kriging function and a spherical semivariogram model (500 m cell size) within the Spatial Analysis Tools. The vessel encounter rate for each grid cell was calculated in ArcMap as the number of vessels sighted divided by the survey effort (km^2^) per cell. Explanatory variables such as habitat type (Fig. 1), water depth, and distance to sanctuary zones, oyster farms, and to land (Supplementary Appendix 1, Fig. S2), were considered fixed in time and included in all models (Table 1). Thus, a single raster layer of water depth was built by pooling *in situ* data collected across the entire study period. Meanwhile, to account for the seasonality of dynamic variables (*i.e*. SST, salinity, water visibility, pH and vessel encounter rate), *in situ* data were pooled per austral season to build seasonal raster layers for each variable (Supplementary Appendix 1, Fig. S3).

#### Ensemble species distribution modelling

To model the presence-absence of dolphins in relation to explanatory variables, we used an ensemble modelling approach that combined results from five different algorithms implemented in Biomod2 package in R v.3.3.2^53^: two regression methods, generalised additive models (GAMs^88^) and generalised boosted models (GBMs^89^); one classification technique, classification tree analysis (CTA^90^); and two machine learning approaches, random forest (RF^91^) and maximum entropy (MaxEnt^92^). These modelling algorithms are known to perform well and provide a good comparison between three different modelling approaches^93,94^. All algorithms were run with the default settings of Biomod2. Within Biomod 2, we used Maxent version 3.4.0, which uses as the default output a complementary log-log (cloglog) transformation to produce an estimate of occurrence probability^95^. Before running the SDMs, correlations between continuous explanatory variables were investigated using correlation coefficients (threshold = 0.7^96^) and variance inflation factors (VIF, threshold = 3^97^). Highly correlated variables were excluded from the set of variables used for SDMs using the stepwise procedures ‘vifcor’ and ‘vifstep’ with the package ‘usdm’ in R^98^. The ‘vifcor’ first finds a pair of variables which has the maximum linear correlation (greater than the threshold), then excludes one of them which has greater VIF; these steps are repeated until there is no variable remaining with a correlation coefficient greater than the threshold. Similarly, ‘vifstep’ first calculates VIF for all variables, then excludes the variable with highest VIF (if this is greater than threshold), and these steps are repeated until no variables with VIF greater than threshold remains^98^.

We built SDMs for the whole study area using data across the entire study period to determine general spatial distribution patterns in relation to benthic habitat type, water depth, and distance to land, sanctuary zones, and oyster farms. Besides, we built seasonal models (austral spring, summer, autumn and winter) to also consider any seasonal shifts due to explanatory variables that varied in space and time over the study period (*i.e*. encounter rate of vessels, SST, salinity, turbidity and pH) (Table 1). Seasons were defined as winter (June–August), spring (September–November), summer (December–February), and autumn (March–May). The response curves of the most important variable of these models indicated that a plateau of high probabilities of dolphins occurred at values within ranges that are only characteristic of the inner area (see results). Previous studies indicated that dolphins in the inner area of Coffin Bay have low emigration rates^37^, strong site fidelity, and most are year-round residents to the inner area^40^. Thus, we also built separate SDMs for the inner area to identify the most important variables influencing the distribution of dolphins residing in this area. Collinearity was explored for each dataset separately.

SDMs were built using a binomial error distribution with logit as the link function. We implemented a 10-fold cross-validation method for each SDM and a random data splitting procedure of 75/25% for respective model calibration and testing using Biomod2^53^. This percentage split of training/testing dataset is a common approach to data partitioning used in various SDM studies (e.g.^36,99–103^), and it is considered best practice for training and testing distribution models^2,104^. The importance of the explanatory variables was assessed using a randomisation procedure in Biomod2 based on 10 permutations^53^. This procedure calculates the Pearson’s correlation between the standard predictions (i.e. fitted values) and predictions where one variable has been randomly permutated, thus allowing direct comparison between models regardless of the modelling method. When the correlation between the two predictions is low, it indicates that the variable is important in the model, and when the correlation is high the variable is not important. The mean correlation coefficient is calculated over multiple runs. The relative importance of each explanatory variable is calculated by subtracting the mean correlation coefficient from 1, so each variable is ranked from zero to one. Variables with zero ranking have no influence in the model, while variables ranked high (closest to one) are considered as the most influential^53^.

The data-splitting procedure allows the evaluation of model accuracy (or predictive performance) when data are non-independent^53^. The area under the receiver operating characteristics curve (AUC) was used to assess SDMs predictive performance and compare SMDs^104^. As non-independent data were used for model evaluation, variability in model accuracy can be interpreted as a measure of the sensitivity of the model results to the initial conditions rather than as a measure of predictive accuracy^53,105^. Here we assumed that models with AUC < 0.7 had poor predictive performance, 0.7 ≤ AUC < 0.9 moderate to good, and AUC ≥ 0.9 excellent performance^105^.

Finally, the five modelling methods were combined to obtain an ensemble prediction of dolphin presence^53^. To generate the ensemble models, only SDMs with AUC ≥ 0.7 were considered and the contribution of selected SDMs to the ensemble model was weighted based on their predictive accuracy^106^. Maps of probability of dolphin occurrence were created based on the ensemble models, where values closer to zero indicate low probabilities, and values closer to one indicate higher probability of presence. When using distribution models to predict occurrence probability of a species to other areas, the values of explanatory variables in the original study area have to be within the ranges of values in the new areas to avoid overestimating the suitability of new areas^2,92^. Since the inner and outer areas of Coffin Bay differ in the ranges of explanatory variables (see Results; Supplementary Appendix 1, Fig. S2), and to avoid making predictions to new collinearity structures in space and/or time^96^, the ensemble predictions of dolphin distribution were done only for the areas corresponding to each dataset (i.e., either the whole Coffin Bay or the inner area only). These included cells where data on explanatory variables was available but had no presence-absence records because of low or null survey effort. Lastly, the performance between the ensemble and single SDMs was compared using AUC values^106^.

To evaluate the relevance of the current zoning of the MPA to the protection of dolphins, the sanctuary zones were overlapped with the predicted values of dolphin occurrence (from the ensemble models), and the mean probability of occurrence (per cell) in each sanctuary zone was estimated.

### Approvals

This study was carried out in accordance to the Flinders University Animal Welfare Committee under the ethics approval of project number E310. Fieldwork was done under Permits to Undertake Scientific Research (numbers: E26171-1, E26171-2, E26171-3 and MR00056-1) from the Department of Environment, Water and Natural Resources (DEWNR), South Australia, and under S115 Ministerial Exemptions (MEs: 9902601, 9902660, 9902714 and 9902779) provided by Primary Industries Resources South Australia (PIRSA).

## Electronic supplementary material

Supplementary Information

## Data Availability

Data made available to all interested researchers upon reasonable request to Cecilia Passadore (cecipass8@gmail.com) and Guido J. Parra (guido.parra@flinders.edu.au).
