# Supplementary material for: Modelling Dolphin Distribution to Inform Future Spatial Conservation Decisions in a Marine Protected Area
Source: Sci Rep. 2018 Oct 23;8:15659. doi: 10.1038/s41598-018-34095-2 (PMC6199262; doi:10.1038/s41598-018-34095-2)
Supplement: Supplementary file 1 — Supplementary Information [file 41598_2018_34095_MOESM1_ESM.pdf]

Modelling dolphin distribution to inform future spatial conservation decisions in a marine protected area

Cecilia Passadore[1,*], Luciana M. Möller[1,2], Fernando Diaz-Aguirre[1,2], Guido J. Parra[1]

[1]Cetacean Ecology, Behaviour and Evolution Lab, College of Science and Engineering, Flinders University, South Australia, 5001, Australia.

[2]Molecular Ecology Lab, College of Science and Engineering, Flinders University, South Australia, 5001, Australia.

[*]Corresponding author

Contact details:

Corresponding author: Cecilia Passadore

Email: cecipass8@gmail.com

Address: Cetacean Ecology, Behaviour and Evolution Lab (CEBEL), College of Science and Engineering, Flinders University, GPO Box 2100, Adelaide, SA 5001, Australia.

Supplementary information

Appendix 1: Spatial and temporal patterns of survey effort, and response and explanatory variables

Between September 2013 and October 2015 survey effort and number of dolphin groups sighted varied between seasons, and between the inner and the outer areas of Coffin Bay (Table S1, Fig. S1). Over 144 days of surveys the transects of the inner and outer areas were covered 39 and 11.5 times, respectively. We encountered 620 groups of dolphins, which corresponded to 587 and 33 in the inner and outer areas, respectively.

Table S1. Summary of boat survey effort conducted in Coffin Bay per season between September 2013 and October 2015 including number of transects surveyed in the inner and outer areas, respectively, number of southern Australian bottlenose dolphin (*Tursiops* cf. a*ustralis*) groups encountered, and number of cells with presences (i.e. cells with survey effort above the mean effort and presence of dolphins) used to model the presence of dolphins.

Study area		Overall	Spring	Summer	Autumn	Winter
Inner	Number of transects surveyed	39	13.5	7.5	11	7
	Number of dolphin groups sighted on effort	587	190	89	155	153
	Number of cells with presences	222	102	64	96	104
Outer	Number of transects surveyed	11.5	3	1.5	5	2
	Number of dolphin groups sighted on effort	33	7	7	13	6
	Number of cells with presences	24	6	3	8	5
Total	Number of dolphin groups sighted on effort	620	197	96	168	159
	Number of cells with presences	246	108	67	104	109

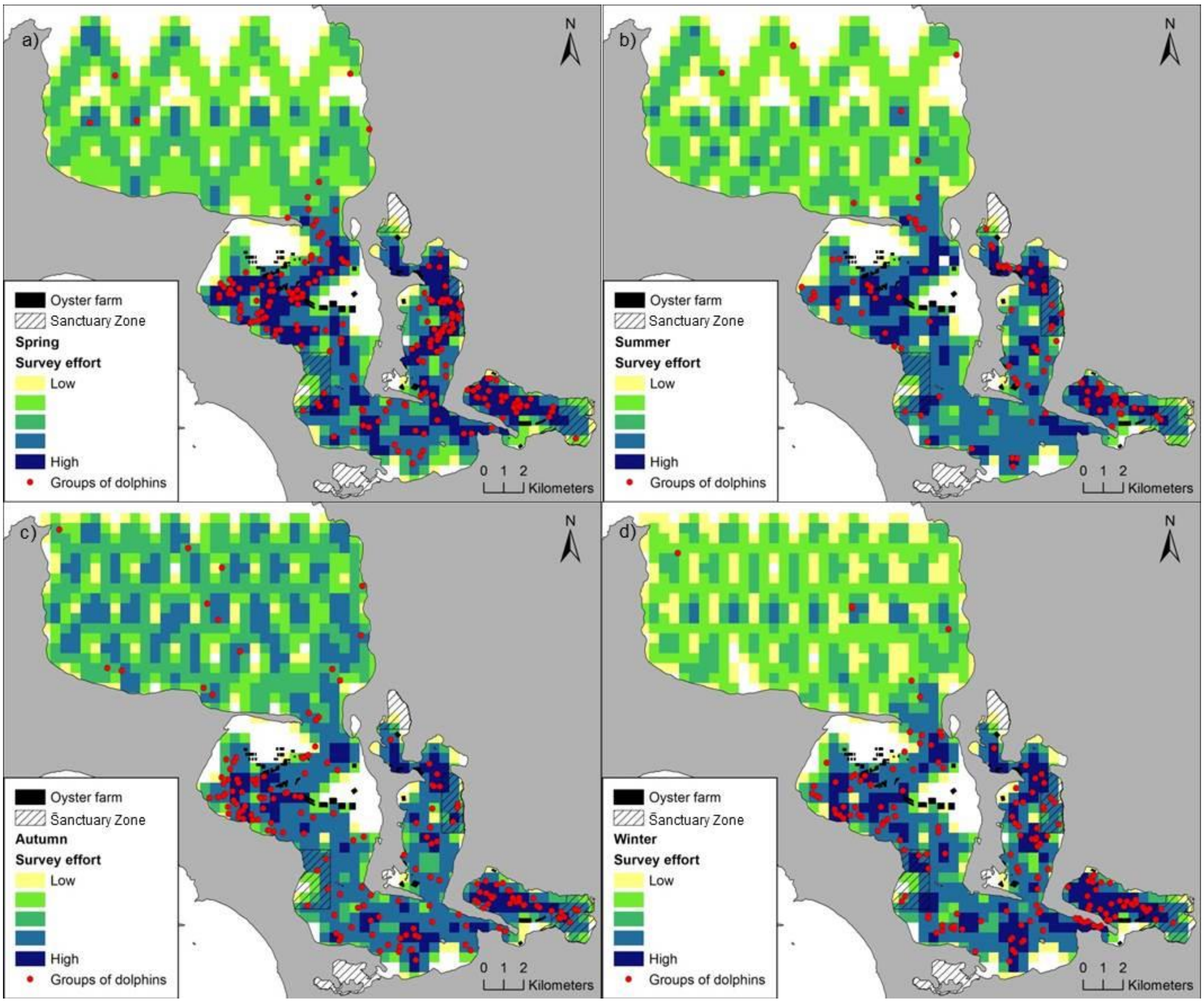

Figure S1. Map of survey area showing survey effort and groups of SABD (*Tursiops* cf. a*ustralis*) encountered during a) spring; b) summer; c) autumn; and d) winter in Coffin bay between September 2013 and October 2015. Cells with values of survey effort above the mean effort per season are indicated with light blue and blue colours; these cells were used to define presence and absences of dolphins to be included in the species distribution models.

The explanatory variables benthic habitat type (Fig. 1), depth, and distance to sanctuary zones, oyster farms, and to land (Fig. S2) were considered fixed in time. In the entire study area, distance to land varied from 0 to 6,756 m; and in the inner area this did not exceeded the 3,000 m (Fig. S2a). Water depth varied from zero to 36 m; with the inner area waters only reaching up to 11 m depth (Fig. S2b). The maximum distance to oyster farms in the outer area of Coffin Bay was 15,558 m, while in the inner area the maximum distance was 5,000 m approx. (Fig. S2c). The maximum distance to sanctuary zones was 21,188 m in the outer area, while in the inner area was less than 5,000 m (Fig. S2d).

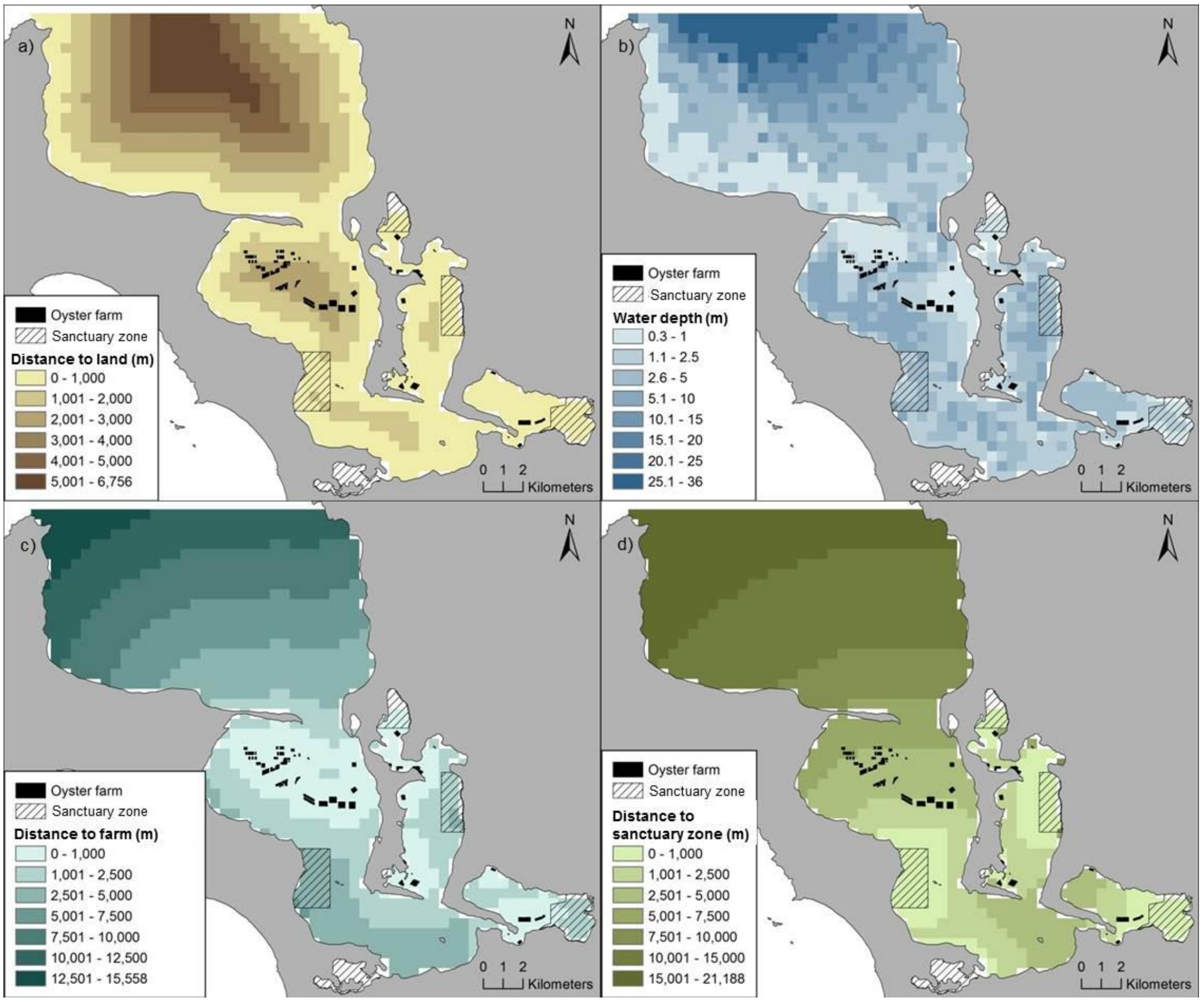

Figure S2. Fixed ecogeographical and anthropogenic variables considered in the modelling of southern Australian bottlenose dolphins (*Tursiops* cf. a*ustralis*) presence in Coffin Bay: a) distance to land; b) depth; c) distance to oyster farms; and d) distance to sanctuary zone.

Spatial and temporal variations were observed in the encounter rate of vessels and ecogeographic variables such as sea surface temperature (SST), salinity, water visibility and pH (Fig. S3). Vessel encounter rates were higher in spring and autumn, and spread over most of the study area (Fig. S3). In winter, SST was lower and relatively homogeneous (12.6 – 14.7 °C) across the study area; in the remaining seasons SST increased from the outer area towards the innermost parts of Coffin Bay, with the highest SST gradient (14.5 – 24.5 °C) recorded during summer and lesser gradients in autumn (15.5 – 19.1 °C) and spring (14.7 – 17.8 °C; Fig. S3). Salinity decreased from outer area towards inner area in winter (37.2 – 33.7 PSU), with lowest values recorded in Kellidie Bay; while the salinity gradient was inverted in the remaining seasons (35.7 – 37.3, 35.6 – 43.4, and 35.2 – 45.1 in spring, summer and autumn, respectively), with the highest values recorded in the innermost embayments (i.e. Kellidie and Mount Dutton Bays; Fig. S3). Spatial patterns of water visibility accompanied the depth profile (Fig. S2b, Fig. S3). The pH tended to increase towards the inner parts of Coffin Bay. Both water visibility and pH were relatively consistent across seasons (Fig. S3).

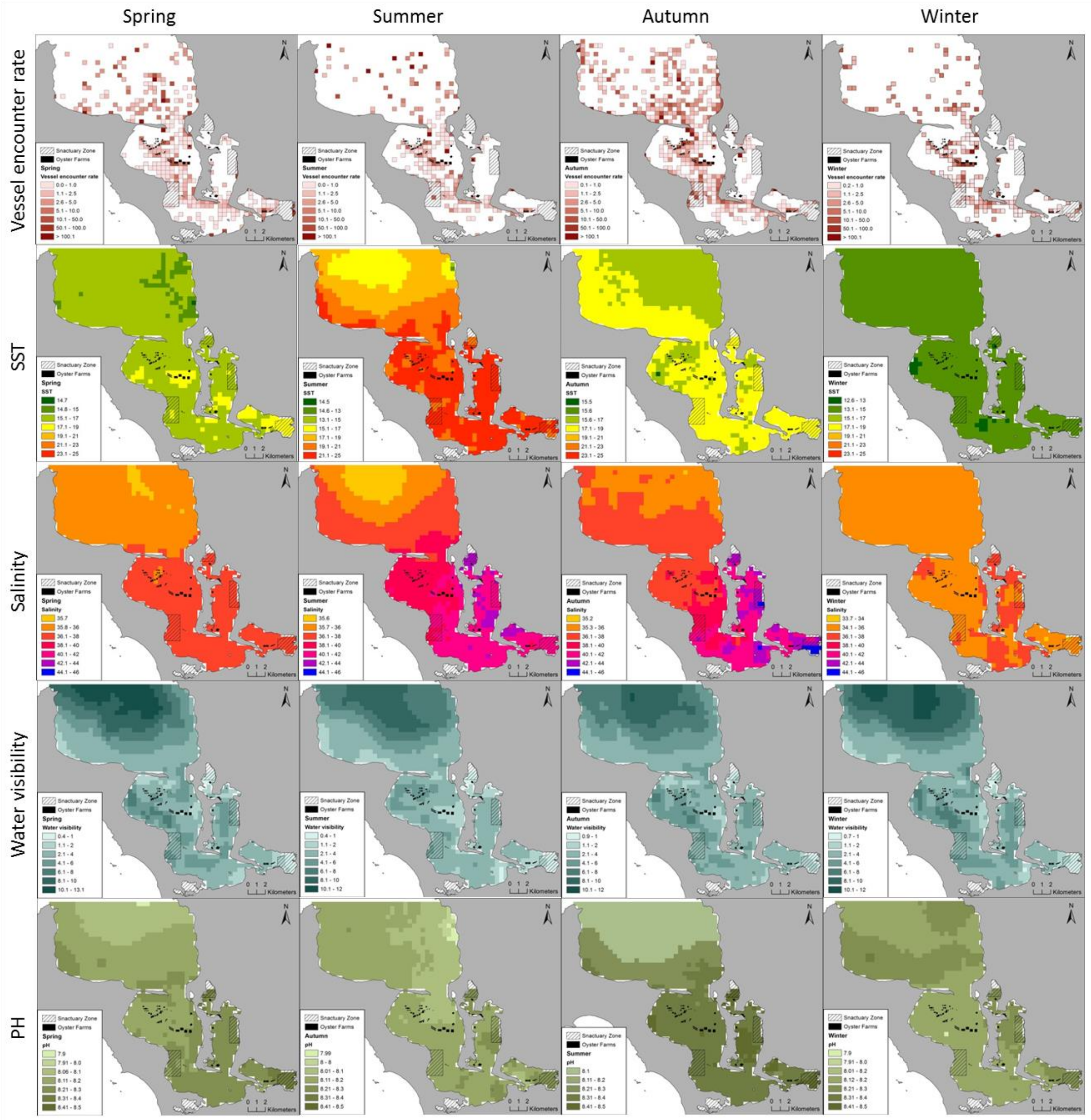

Figure S3. Ecogeographical and anthropogenic variables considered in the modelling of southern Australian bottlenose dolphins (*Tursiops* cf. a*ustralis*) presence in Coffin Bay by season. Columns from left to right correspond to: austral spring, summer, autumn, and winter. Each line corresponds to a variable, from top to bottom: encounter rate of vessels, sea surface temperature (SST), salinity, water visibility, and pH.

Appendix 2: Response curves of species distribution models for the overall study period

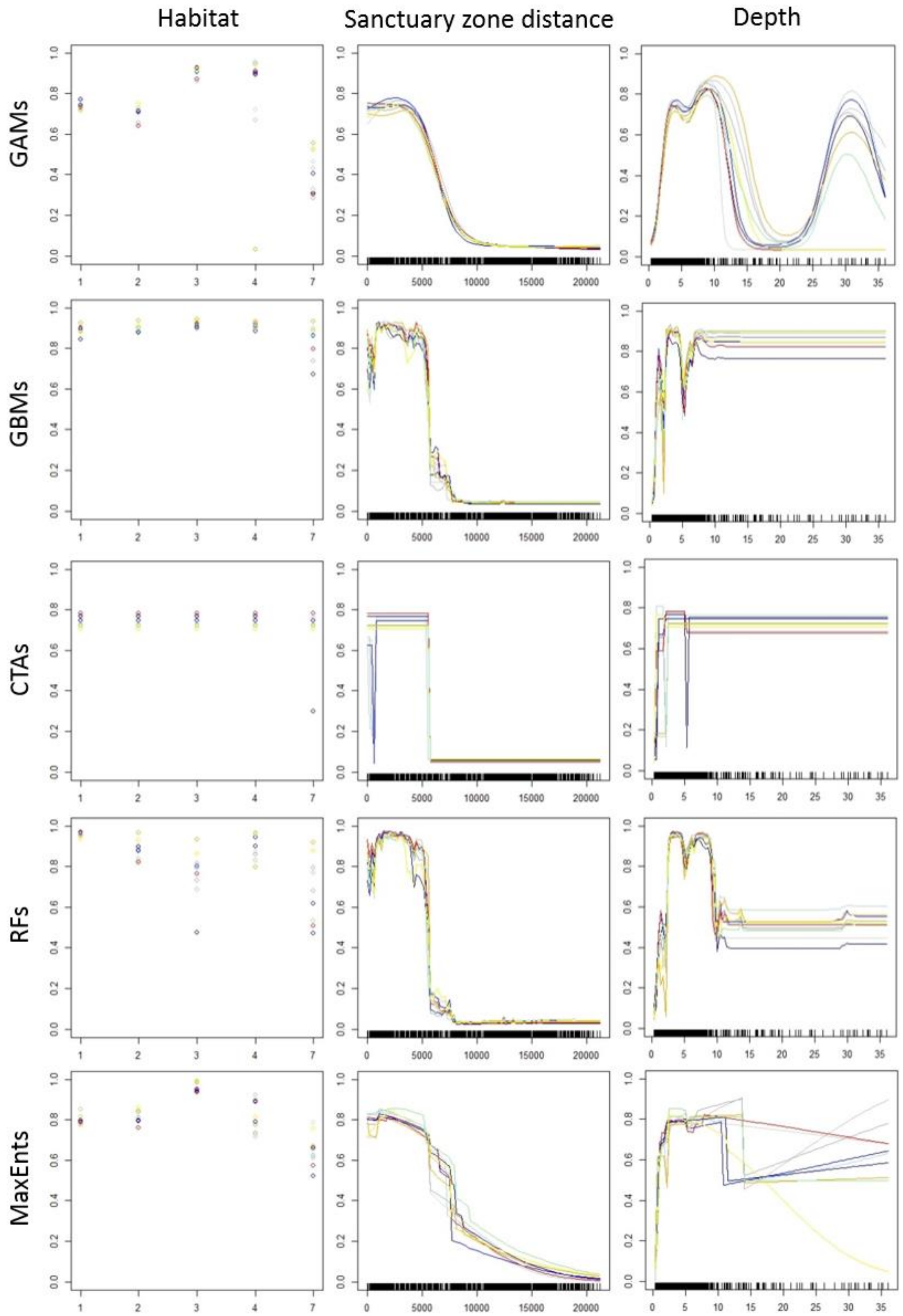

Figure S4. Response curves of presence of dolphins in relation to the explanatory variables obtained for species distribution models run for the entire study area over the entire study period (September 2013 – October 2015). Panes from top to bottom show the curves for each modelling algorithm (GAM, generalised additive model; GBM, generalised boosted model; CTA, classification tree analysis; RF, random forest; and MaxEnt, maximum entropy), and from left to right the explanatory variables (habitat type, distance to sanctuary zone, and depth). Each coloured line represents one of the 10 cross-validation runs.

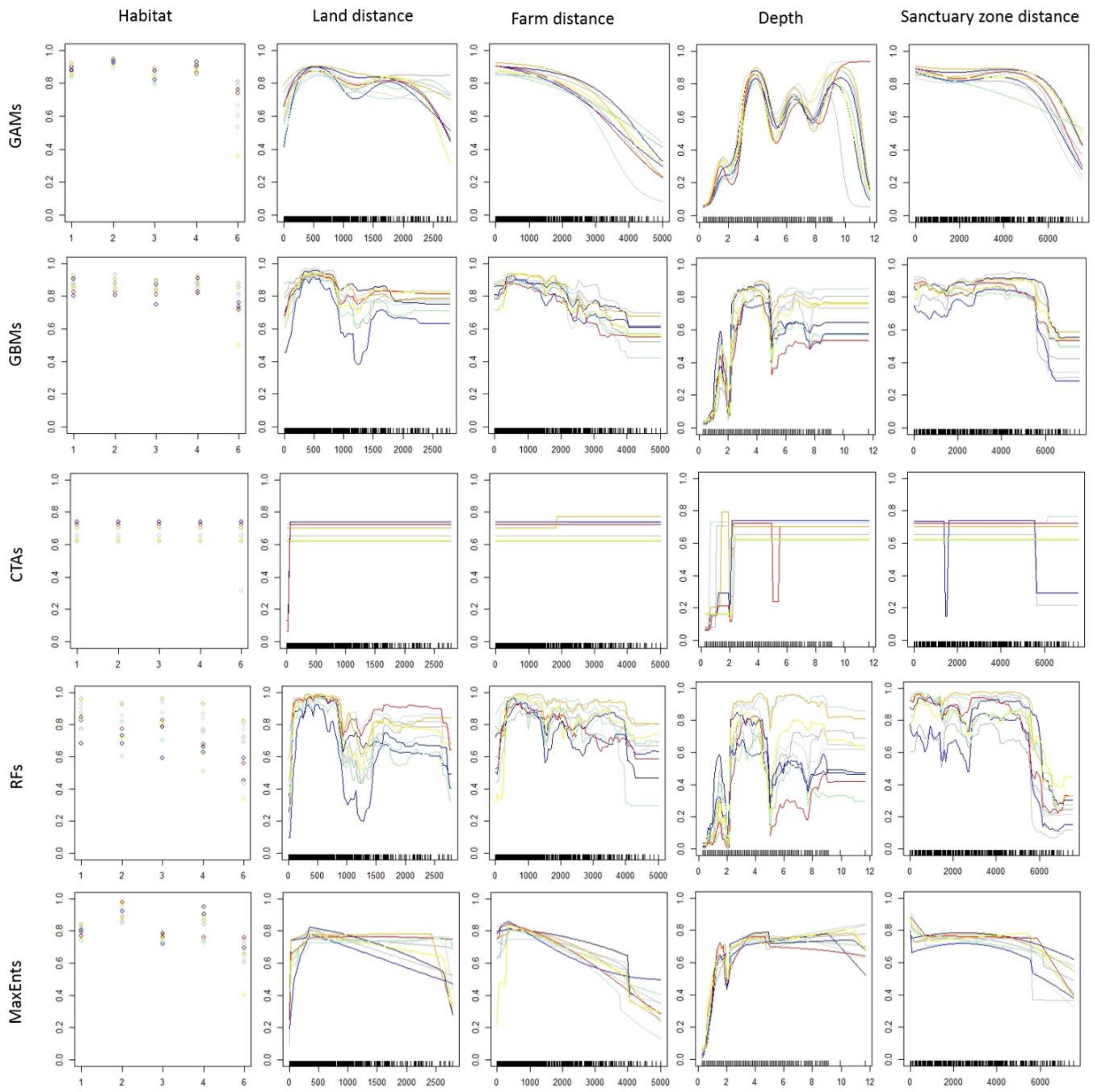

Figure S5. Response curves of presence of dolphins in relation to the explanatory variables obtained for species distribution models run for the inner area of Coffin Bay over the entire study period (September 2013 – October 2015). Panes from top to bottom show the curves for each modelling algorithm (GAM, generalised additive model; GBM, generalised boosted model; CTA, classification tree analysis; RF, random forest; and MaxEnt, maximum entropy), and from left to right the explanatory variables (habitat, distance to land, distance to oyster farm, depth, and distance to sanctuary zone). Each coloured line represents one of the 10 cross-validation runs.

Appendix 3: Performance and response curves of the seasonal species distribution models for the inner area of Coffin Bay

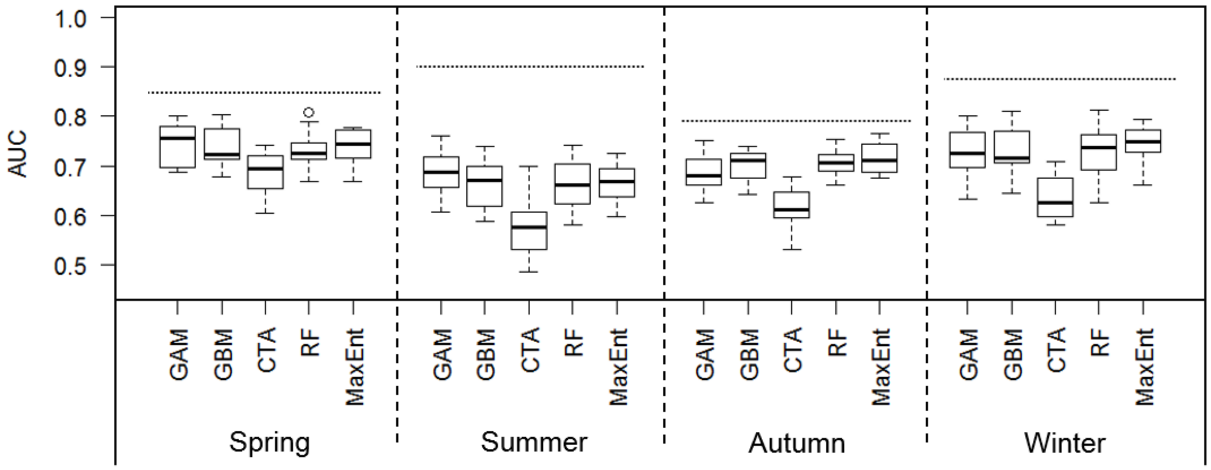

Figure S6. Performance of species distribution models built with seasonal (i.e. spring, summer, autumn, winter) datasets of the inner area of Coffin Bay. Box-plots for the model accuracy (AUC: area under the curve of the receiver operating characteristics plot) of the 10 cross-validation runs of each modelling algorithm (GAM: generalised additive model; GBM: generalised boosted model; CTA: classification tree analysis; RF: random forest; and MaxEnt: maximum entropy). Dotted lines indicate the predictive performance (AUC) of ensemble models for each dataset (AUC was 0.85, 0.90, 0.79 and 0.88 in spring, summer, autumn and winter, respectively). Values of AUC ≥ 0.7 indicate that the model predictive performance is moderate to excellent.

The response curves of presence of dolphins in relation to the explanatory variables obtained for seasonal species distribution models run for the inner area of Coffin Bay are presented in Fig. S7.

Figure S7. Response curves of presence of dolphins in relation to the explanatory variables obtained for seasonal species distribution models run for the inner area of Coffin Bay. Panes are presented ordered by austral season (Spring, Summer, Autumn, and Winter) are grouped per modelling algorithm (GAM, generalised additive model; GBM, generalised boosted model; CTA, classification tree analysis; RF, random forest; and MaxEnt, maximum entropy). Each coloured line represents one of the 10 cross-validation runs.

Spring SDMs' response curves:

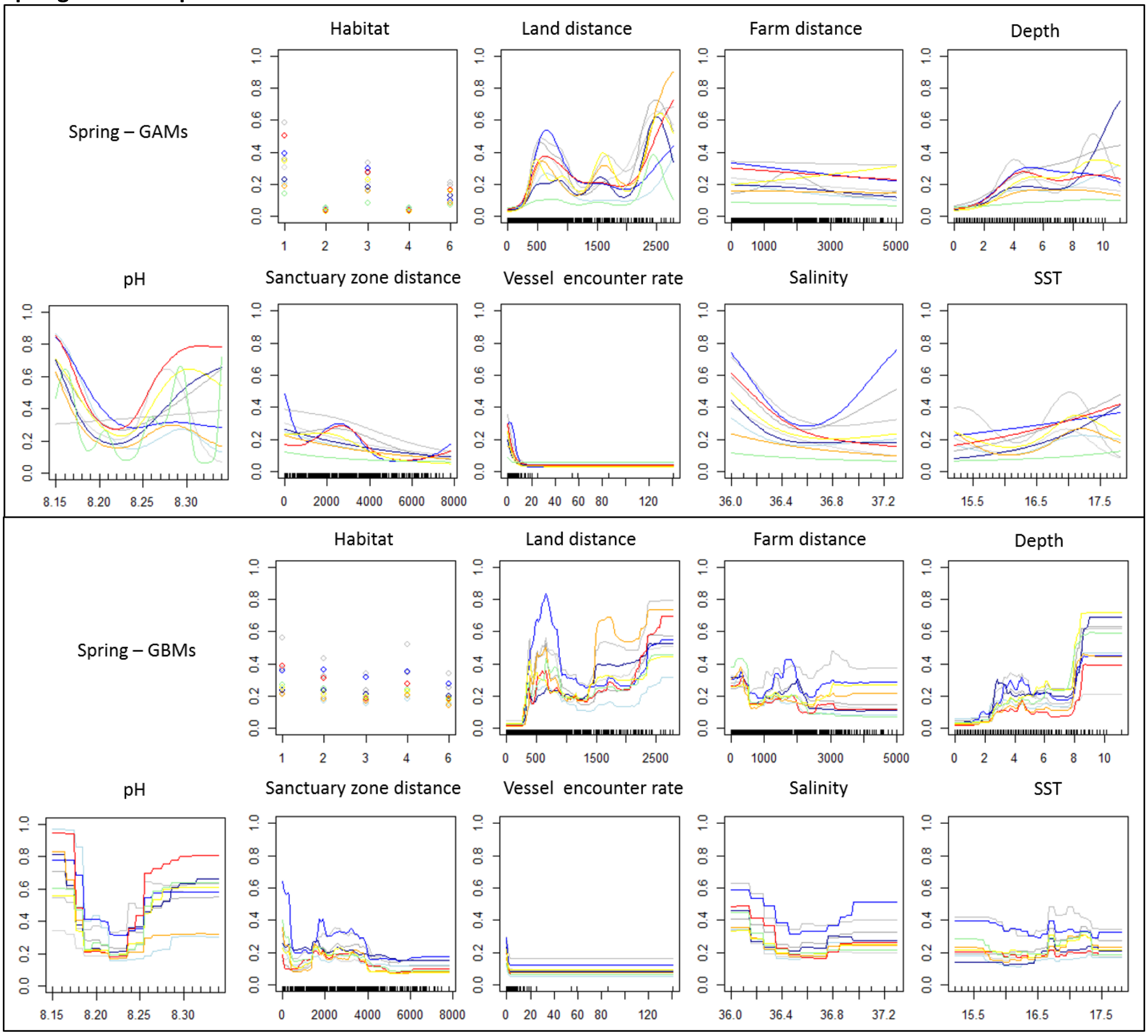

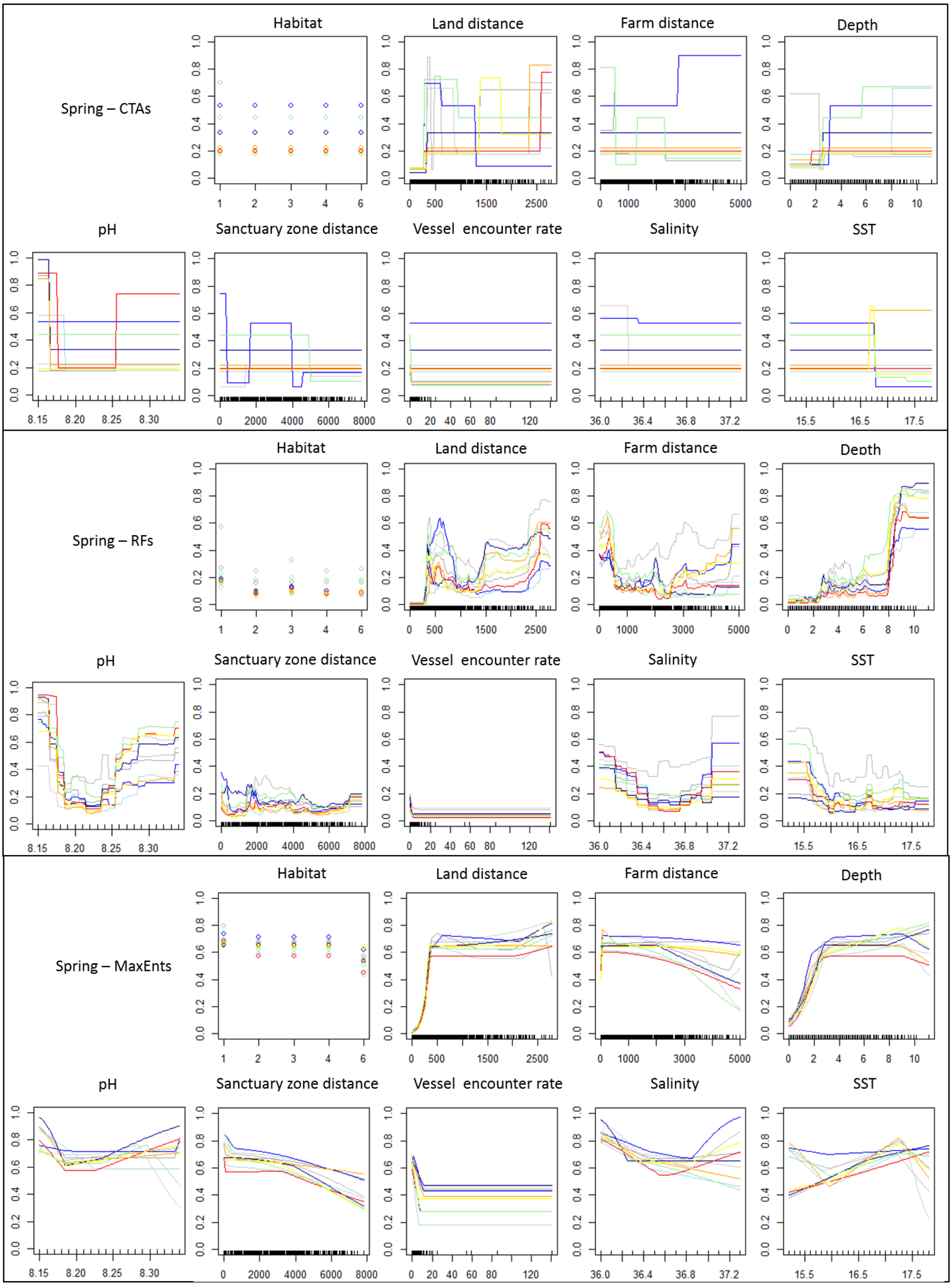

Spring – CTAs
Spring – RFs
Spring – MaxEnts
Habitat
Land distance
Farm distance
Depth
pH
Sanctuary zone distance
Vessel encounter rate
Salinity
SST

Summer SDMs' response curves:

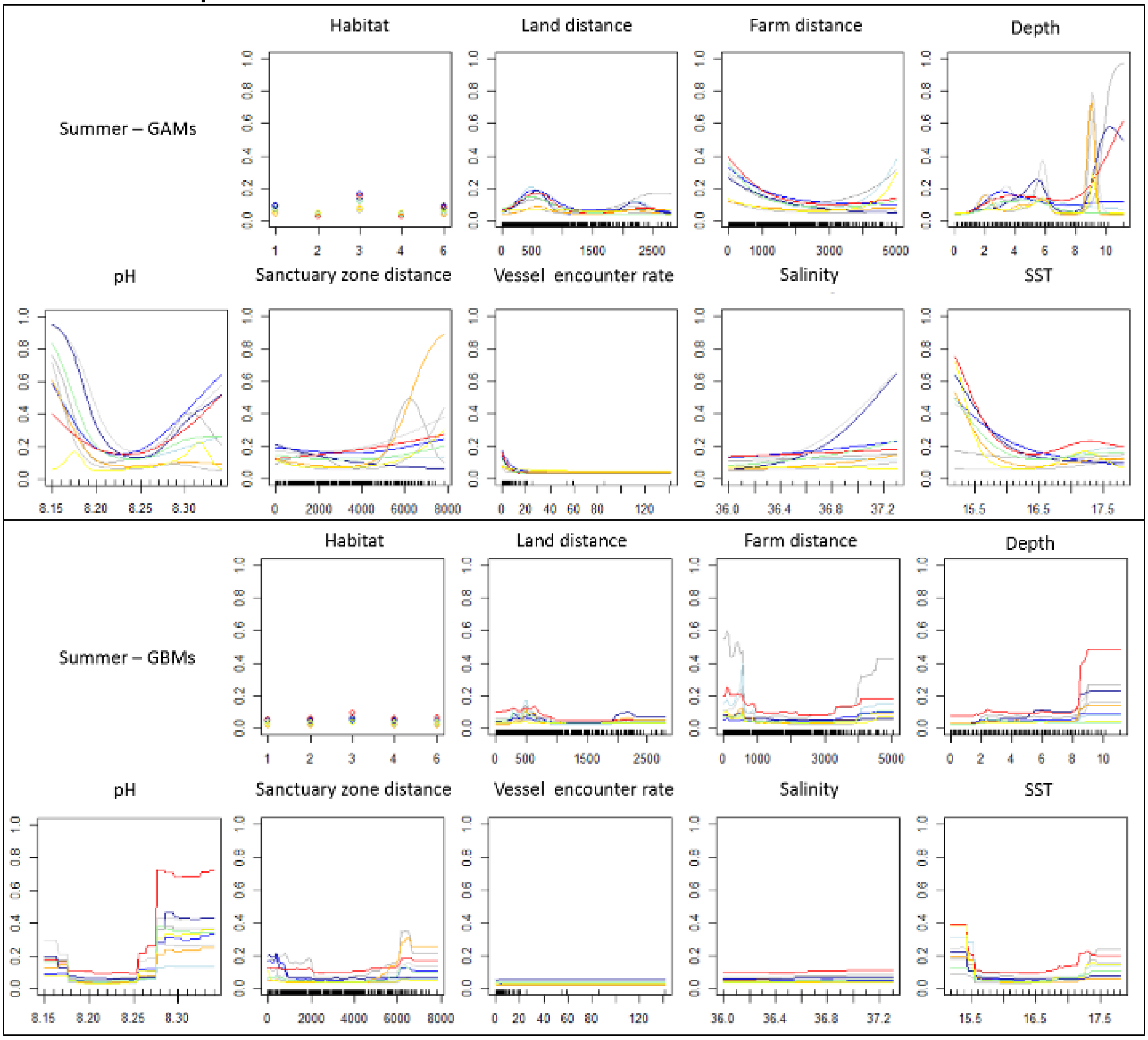

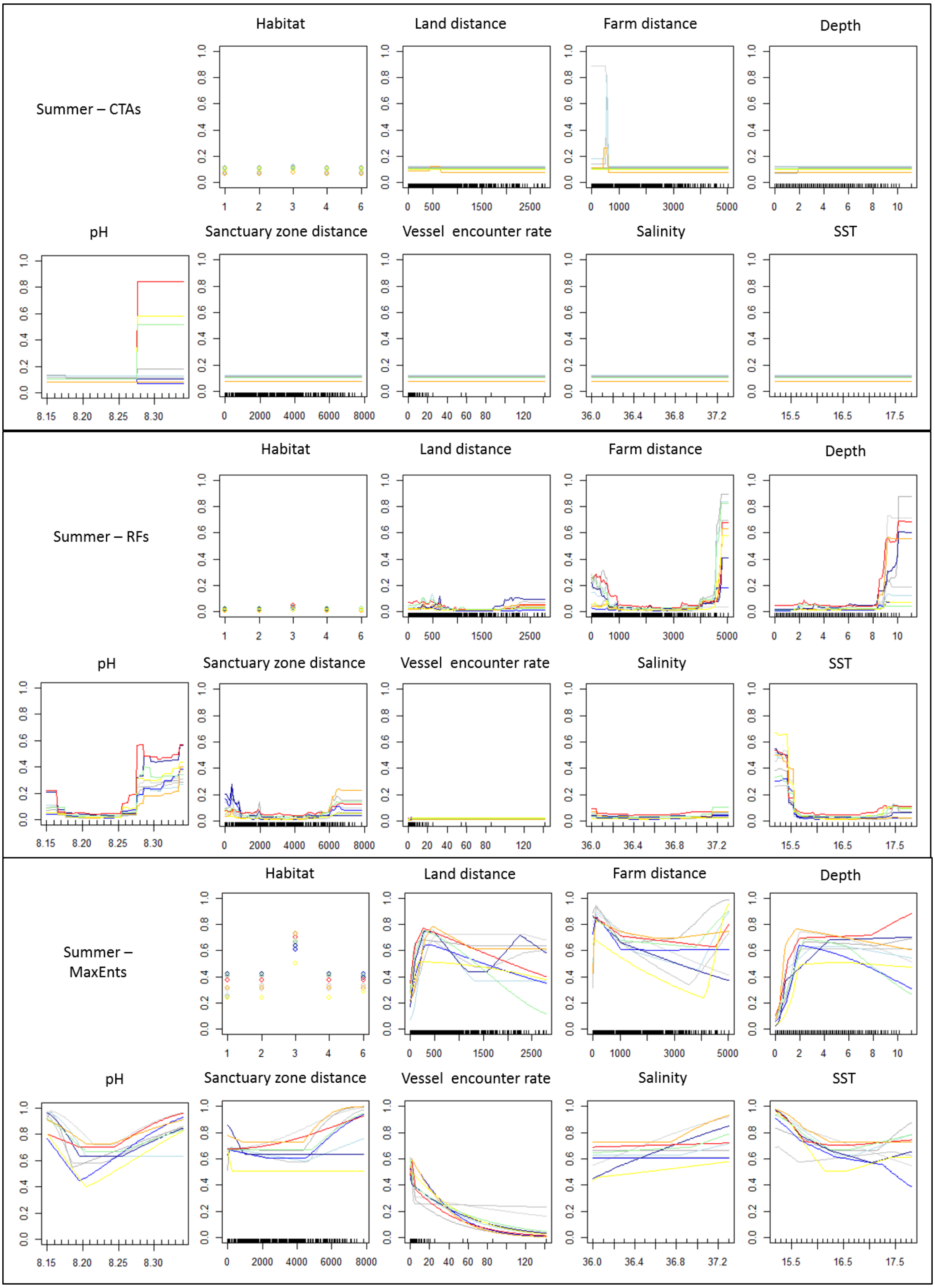

Summer – CTAs
Habitat
Land distance
Farm distance
Depth
pH
Sanctuary zone distance
Vessel encounter rate
Salinity
SST
Summer – RFs
Habitat
Land distance
Farm distance
Depth
pH
Sanctuary zone distance
Vessel encounter rate
Salinity
SST
Summer – MaxEnts
Habitat
Land distance
Farm distance
Depth
pH
Sanctuary zone distance
Vessel encounter rate
Salinity
SST

Autumn SDMs' response curves:

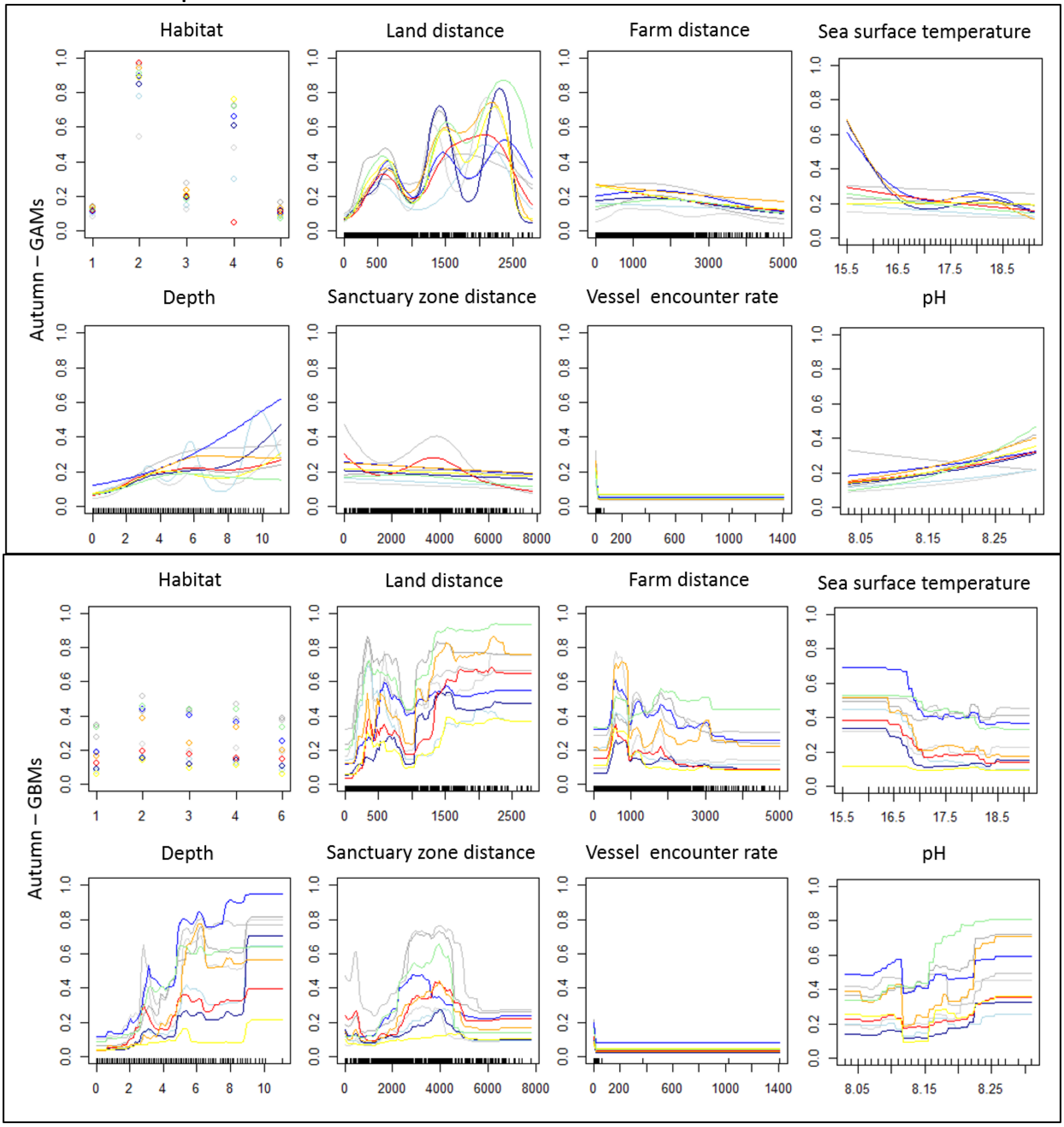

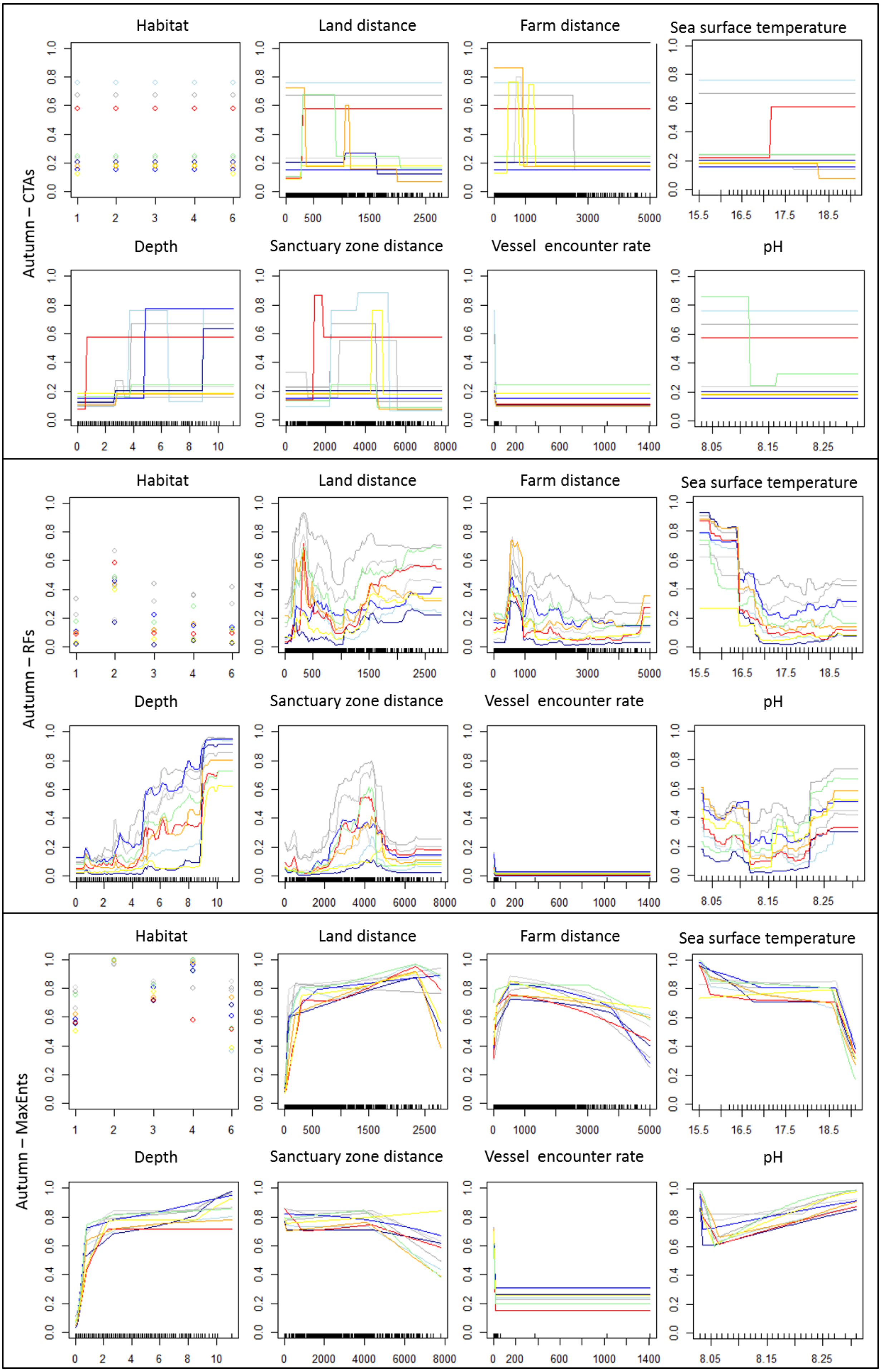

Autumn – CTAs
Autumn – RFs
Autumn – MaxEnts
Habitat
Land distance
Farm distance
Sea surface temperature
Depth
Sanctuary zone distance
Vessel encounter rate
pH

Winter SDMs' response curves:

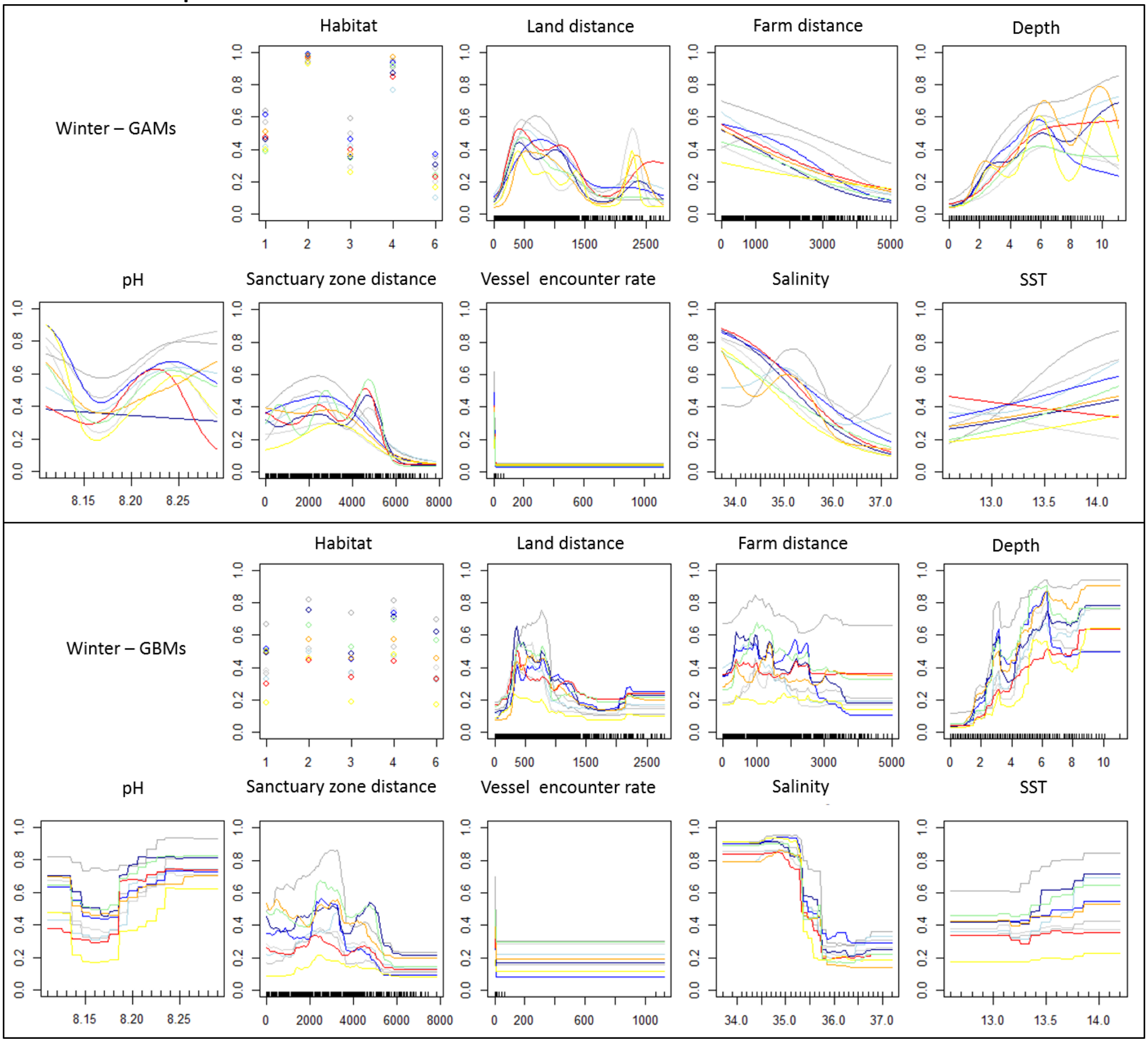

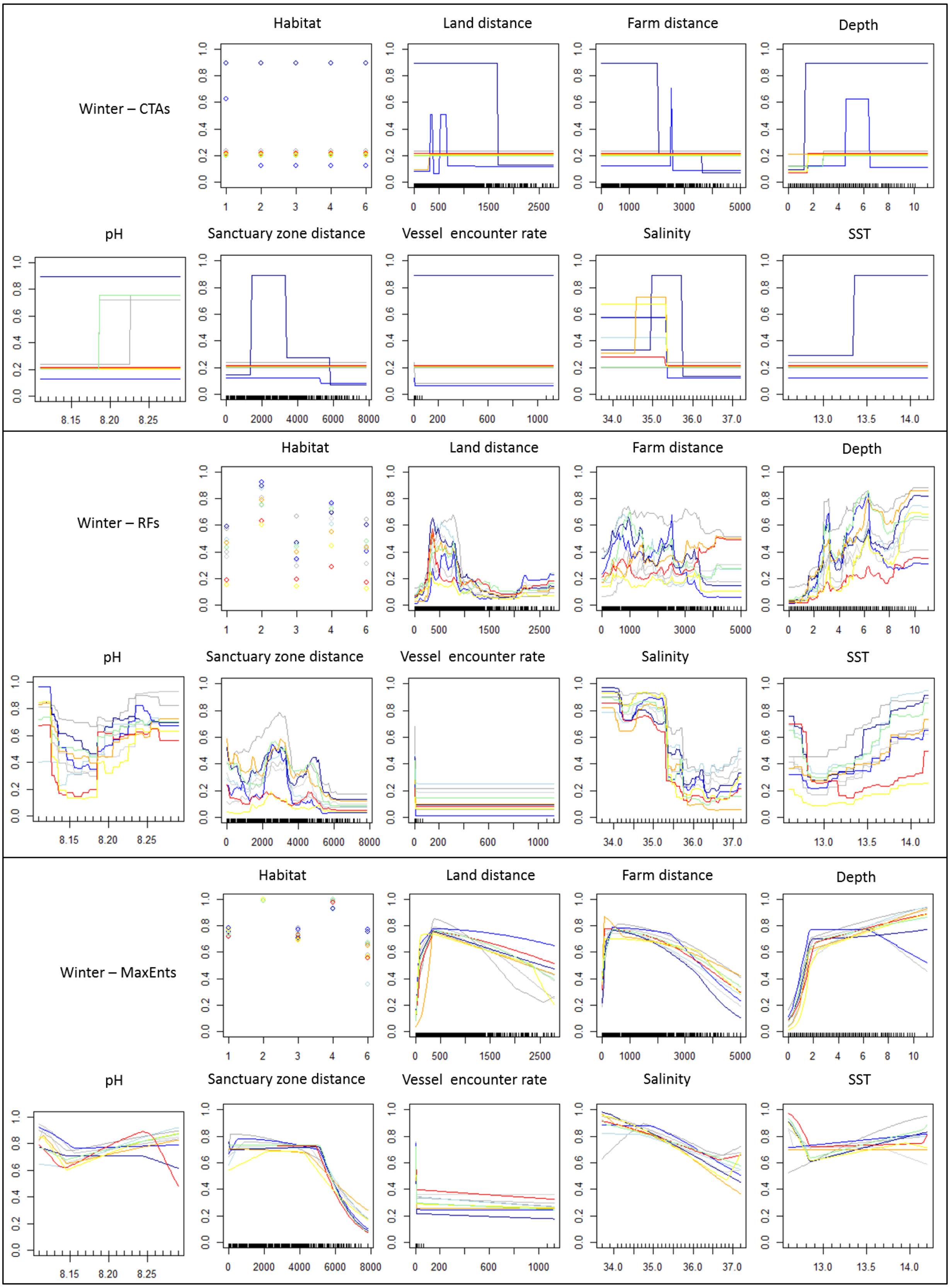

Winter – CTAs
Winter – RFs
Winter – MaxEnts
Habitat
Land distance
Farm distance
Depth
pH
Sanctuary zone distance
Vessel encounter rate
Salinity
SST

Appendix 4: Seasonal probability of dolphin occurrence in the entire study area

For the seasonal datasets of the entire Coffin Bay, collinearity was found between several explanatory variables originally considered to model the presence of dolphins. Some variables were discarded from modelling after running 'vifcor' (threshold = 0.7) and 'vifstep' (threshold = 3) (see variables included in models in Table S2). For every season, most single SDMs of the entire study area showed from moderate to good predictive performance (0.7 ≤ AUC < 0.9), except for some CTAs that had poor (AUC < 0.7) performance (Fig. S8). In spring, autumn and winter, the performance of ensemble models was better than the performance of all single SDMs (Fig. S8). While in summer the ensemble model outperformed most single SDMs runs, a few single SDM runs had better performance than the ensemble predictions (Fig. S8). In general, the most important variables related with the presence of dolphins per season were either distance to oyster farms (in spring, summer and winter) or distance to sanctuary zones (in autumn) instead of ecogeographic variables that change along the year such as vessels encounter rate, salinity or SST (Table S2). In general, the response curves of seasonal SDMs during these seasons indicated that the probability of dolphin occurrence is higher in cells closer to farms (less than 5000 m) and in shallower areas (less than 10 m).

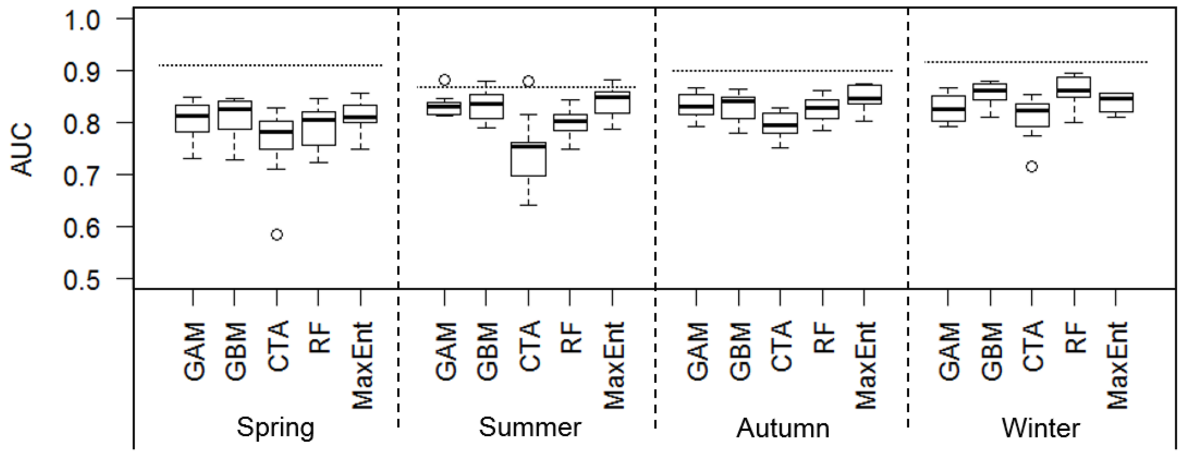

Figure S8. Box-plots for the model accuracy (AUC: area under the curve of the receiver operating characteristics plot) of the 10 cross-validation runs of each single species distribution model (GAM: generalised additive model; GBM: generalised boosted model; CTA: classification tree analysis; RF: random forest; and MaxEnt: maximum entropy). Dotted lines indicate the predictive performance (AUC) of ensemble models for each dataset (AUC was 0.91, 0.87, 0.90 and 0.92 in spring, summer, autumn and winter, respectively). Values of AUC ≥ 0.7 indicate that the model predictive performance is moderate to excellent.

Table S2. Importance of ecogeographical and anthropogenic variables for bottlenose dolphins (*Tursiops* cf. *australis*) in the entire Coffin Bay per season, using five types of models: generalised additive model (GAM), generalised boosted model (GBM), classification tree analysis (CTA), random forest (RF) and maximum entropy (MaxEnt). Variable importance is presented as the mean value over the 10 runs of each single modelling algorithm, and as the mean of means amongst them. Ecogeographical variables of greatest influence (values closest to 1) are highlighted in bold. (NOTE: Values are presented only for the variables included in models)

Season	Model	Habitat type	Distance to sanctuary zone	Water depth	Distance to land	Distance to oyster farm	Vessel encounter rate	Salinity	Sea surface temperature	pH
Spring	GAM	0.115	--	--	0.348	**0.552**	0.043	--	0.409	--
	GBM	0.005	--	--	0.336	**0.770**	0.087	--	0.316	--
	CTA	0.044	--	--	0.524	**0.819**	0.167	--	0.276	--
	RF	0.028	--	--	0.313	**0.630**	0.142	--	0.349	--
	MaxEnt	0.012	--	--	0.117	**0.556**	0.015	--	0.223	--
	Mean of means	0.041	--	--	0.328	**0.666**	0.091	--	0.314	--
Summer	GAM	0.250	--	--	0.379	**0.743**	0.132	--	--	--
	GBM	0.149	--	--	0.475	**0.834**	0.039	--	--	--
	CTA	0.200	--	--	0.471	**0.911**	0.030	--	--	--
	RF	0.140	--	--	0.517	**0.798**	0.061	--	--	--
	MaxEnt	0.104	--	--	0.121	**0.790**	0.044	--	--	--
	Mean of means	0.168	--	--	0.393	**0.815**	0.061	--	--	--
Autumn	GAM	0.142	**0.566**	0.495	--	0.284	0.115	--	0.017	0.075
	GBM	0.015	**0.614**	0.447	--	0.187	0.100	--	0.027	0.048
	CTA	0.019	**0.868**	0.563	--	0.059	0.061	--	0.013	0.103
	RF	0.020	**0.317**	0.308	--	0.211	0.101	--	0.088	0.070
	MaxEnt	0.023	**0.403**	0.246	--	0.327	0.100	--	0.013	0.038
	Mean of means	0.044	**0.554**	0.412	--	0.214	0.095	--	0.032	0.067
Winter	GAM	0.111	--	--	0.404	**0.800**	0.051	0.183	--	0.121
	GBM	0.013	--	--	0.331	**0.784**	0.048	0.182	--	0.137
	CTA	0.057	--	--	0.418	**0.746**	0.019	0.367	--	0.220
	RF	0.039	--	--	0.310	**0.633**	0.068	0.197	--	0.175
	MaxEnt	0.020	--	--	0.215	**0.840**	0.035	0.023	--	0.022
	Mean of means	0.048	--	--	0.335	**0.761**	0.044	0.190	--	0.135

The ensemble models per season using data of the entire study area predicted higher probabilities of dolphins' presence consistently in the inner area of Coffin Bay (Fig. S9), where the distance to oyster farms and to sanctuary zones is less than 5,000 m (Fig. S2c, d). Summer prediction showed the lowest probability of presence of dolphins compared with the rest of the seasons, the highest probability of dolphins in summer were in Kellidie bay and the northern part of Mount Dutton bay, including waters of Little Mount Dutton (Fig. S9b). In the remaining seasons, the highest probability of dolphins presence were predicted in the western sector of Kellidie bay, where water is deeper than 2 m, in Mount Dutton bay and some parts of Port Douglas bay, particularly close to the oyster farms where water is deeper than 1 m (Fig. S9a, c, d).

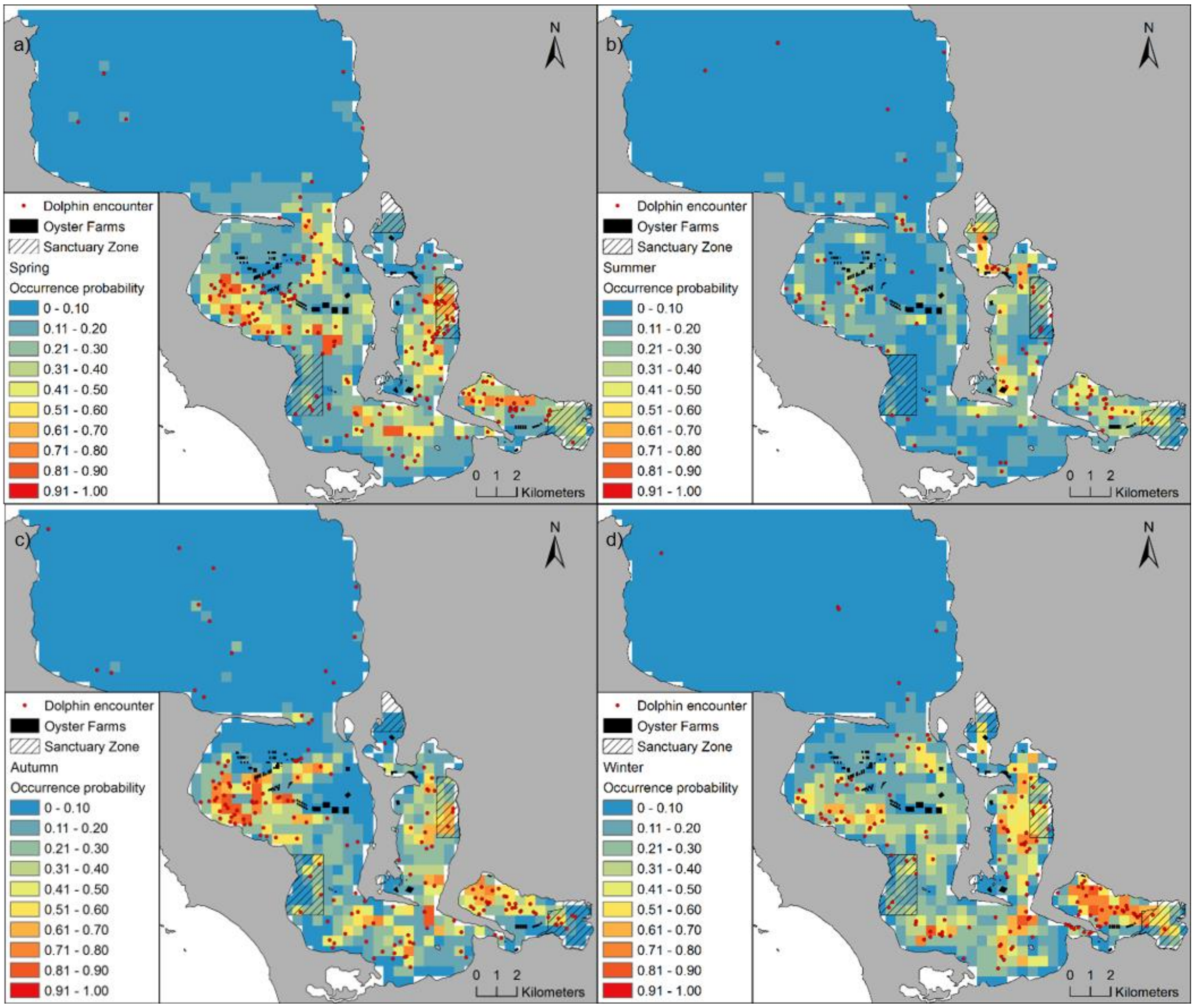

Figure S9. Ensemble model prediction of the probability of occurrence of southern Australian bottlenose dolphin in Coffin Bay per season: a) spring; b) summer; c) autumn; and d) winter. The coloured shading, as detailed in the legend, represents probability of dolphin occurrence.
